# Pinolenic acid exhibits anti-inflammatory and anti-atherogenic effects in peripheral blood-derived monocytes from patients with rheumatoid arthritis

**DOI:** 10.1038/s41598-022-12763-8

**Published:** 2022-05-25

**Authors:** Rabaa Takala, Dipak P. Ramji, Robert Andrews, You Zhou, Mustafa Farhat, Mohammed Elmajee, Shelley Rundle, Ernest Choy

**Affiliations:** 1 Division of Infection and Immunity, School of Medicine, Cardiff University, Cardiff, UK; 2 Cardiff School of Biosciences, Cardiff University, Cardiff, UK; 3 Systems Immunity University Research Institute, Cardiff University, Cardiff, UK; 4 Department of Immunology and Immunotherapy, School of Cancer Sciences, University of Birmingham, Birmingham, UK; 5 Orthopaedic Department, University Hospitals Birmingham NHS Foundation Trust, Birmingham, UK; 6 Wales Gene Park, Division of Cancer and Genetics, School of Medicine, Cardiff University, Cardiff, UK; 7 Rheumatology, University Hospital of Wales, Cardiff, UK

**Keywords:** Drug discovery, Genetics, Immunology, Rheumatology

## Abstract

Pinolenic acid (PNLA), an omega-6 polyunsaturated fatty acid from pine nuts, has anti-inflammatory and anti-atherogenic effects. We aimed to investigate the direct anti-inflammatory effect and anti-atherogenic effects of PNLA on activated purified CD14 monocytes from peripheral blood of patients with rheumatoid arthritis (RA) in vitro. Flow cytometry was used to assess the proportions of CD14 monocytes expressing TNF-α, IL-6, IL-1β, and IL-8 in purified monocytes from patients with RA after lipopolysaccharide (LPS) stimulation with/without PNLA pre-treatment. The whole genomic transcriptome (WGT) profile of PNLA-treated, and LPS-activated monocytes from patients with active RA was investigated by RNA-sequencing. PNLA reduced percentage of monocytes expressing cytokines: TNF-α by 23% (p = 0.048), IL-6 by 25% (p = 0.011), IL-1β by 23% (p = 0.050), IL-8 by 20% (p = 0.066). Pathway analysis identified upstream activation of peroxisome proliferator-activated receptors (PPARs), sirtuin3, and let7 miRNA, and KLF15, which are anti-inflammatory and antioxidative. In contrast, DAP3, LIF and STAT3, which are involved in TNF-α, and IL-6 signal transduction, were inhibited. Canonical Pathway analysis showed that PNLA inhibited oxidative phosphorylation (p = 9.14E−09) and mitochondrial dysfunction (p = 4.18E−08), while the sirtuin (SIRTs) signalling pathway was activated (p = 8.89E−06) which interfere with the pathophysiological process of atherosclerosis. Many miRNAs were modulated by PNLA suggesting potential post-transcriptional regulation of metabolic and immune response that has not been described previously. Multiple miRNAs target pyruvate dehydrogenase kinase-4 (PDK4), single-immunoglobulin interleukin-1 receptor molecule (SIGIRR), mitochondrially encoded ATP synthase membrane subunit 6 (MT-ATP6) and acetyl-CoA acyltranferase2 (ACAA2); genes implicated in regulation of lipid and cell metabolism, inflammation, and mitochondrial dysfunction. PNLA has potential anti-atherogenic and immune-metabolic effects on monocytes that are pathogenic in RA and atherosclerosis. Dietary PNLA supplementation regulates key miRNAs that are involved in metabolic, mitochondrial, and inflammatory pathways.

## Introduction

RA is an autoimmune inflammatory disease that causes chronic joint inflammation. Cardiovascular diseases (CVDs) are the leading cause of death in patients with RA due to accelerated atherosclerosis. Monocytes/macrophages cause synovitis and produce pro-inflammatory cytokines. They are also important in atherosclerosis, as the most abundant immune cells within plaques and orchestrate the initiation, progression, and destabilisation of these lesions. Despite the increasing number of new treatments in RA, most patients do not achieve clinical remission, and residual pain and disability are common^1^. Moreover, side effects and safety concerns of anti-rheumatic medications lead to poor adherence^2^. A healthy and balanced diet including omega(n)-3 and other polyunsaturated fatty acid (PUFA) is recommended by American College of Rheumatology (ACR) and European Alliance of Associations for Rheumatology (EULAR) to reduce the risk of RA. However, there is no recommendation on specific type of food due to limited scientific evidence.

PNLA is an n-6 PUFA found exclusively in pine nuts oil (*Pinus orientalis*) and maritime pine (*Pinus pinaster*) seed oils, so pine nuts are a rich source of PNLA^3–5^. According to Nutritional Science, the richest source of PNLA is the oil pressed from Siberian Pine Nuts, which contains up to 27% of this PUFA. The Korean Pine Nuts are another good source of PNLA making up to 20% of its content.

Initial studies showed that PNLA has anti-inflammatory and antioxidant actions in cell lines and animal models^3–5^. Recent studies of PNLA on RA patients’ peripheral blood (PB) samples identified several anti-inflammatory and potential lipid lowering effects^6^. Takala et al. in 2021 have demonstrated that PNLA significantly reduces monocyte migration, lipid uptake and macropinocytosis in THP-1 and primary cultures of macrophages in-vitro and ex-vivo^6^. They also demonstrated that PNLA produces a significant reduction in the levels of TNF-α, IL-6, and PGE2 in supernatants of lipopolysaccharide (LPS) activated PBMCs from RA patients and healthy controls (HCs). No difference was observed between RA patients and HCs even though cytokine levels are higher in RA patients^6^. Previous transcriptomic work on PBMCs found that PNLA regulates the expression of metabolic genes, including PDK4, FBP1, and SERPINE1. Moreover, bioinformatic analyses identified that PNLA inhibits NF-κB, STAT1, IL-1 and CCR2, while activates PPARs^6^. These data suggest that PNLA has potential anti-inflammatory and metabolic effects on activated PBMCs from RA patients and HCs.

In the current study, we aimed to confirm and extend the previous findings^6^ and focus on the anti-inflammatory and anti-atherogenic effects of PNLA on purified monocytes from RA patients with active disease. First, the proportion of CD14^**+**^ monocytes expressing pro-inflammatory cytokines TNF-α, IL-6, IL-β, IL-18 and the overall count of activated monocytes with or without PNLA treatment was assessed by flow cytometry. Second, whole genomic transcriptome (WGT) of PNLA treated LPS activated monocytes was assessed.

## Methods

### RA Patients recruitment and PBMCs isolation

Participants at least 35 years old with RA (n = 20; mean age = 62 years) were recruited for assessing the intracellular levels of pro-inflammatory cytokines (TNF-α, IL-6, IL-1β and IL-8) expressed by purified monocytes after LPS-stimulation with or without PNLA. While for the transcriptome study, 8 ACPA positive RA patients, aged from 55 to 70 years old (mean age 61.5 years) and disease duration between 11-22 years with active disease (DAS28 score range 4.8–5.7) were recruited. They were on biologic disease modifying anti-rheumatic drugs with exclusion including pregnancy, active infection, current malignancy and patients with untreated anaemia, and other chronic inflammatory arthritis apart from RA. Detailed demographic and laboratory data are described in Supplementary Tables S1, S2 for intracellular cytokines and WGT assessment, respectively. All patients were recruited from the Rheumatology Department at the University Hospital of Wales, signed informed consent was obtained from all participants. The study was approved by the Research Ethics Committee for Wales 3 (reference no. 12/WA/0045) University Hospital of Wales. PBMCs were isolated by standard Ficoll density gradient centrifugation and methodological details are provided in (Supplementary Data S1). All methods were performed in accordance with the relevant guidelines and regulations.

### Negative selection and sorting of purified monocytes

To purify monocytes for assessment of intracellular cytokines using flow cytometry, monocytes were recovered by negative selection in cascades by magnetic-activated cell sorting (MACS) pan monocyte isolation kit (Miltenyi Biotec, UK) following the manufacturer’s protocol. Pan monocyte isolation kit (Miltenyi Biotec, UK) uses a cocktail of biotinylated antibodies to select non-CD14^**+**^ cells, these cells are magnetically labelled using anti-biotin microbeads and separated from target CD14^**+**^ monocytes cells using a magnetic column. 1 × 10^7^ PBMCs were re-suspended in 100 µl MACS (0.5% BSA, 5 mM EDTA, and 0.09% sodium azide in PBS), and 10 µl of FcR blocking reagent and 10 µl of biotin-antibody cocktail (provided in the kit) were added. The suspension was then mixed and incubated for 10 min at 2–8 °C. A 20 µl MACS buffer and 20 µl of anti-biotin microbeads were added for1 × 10^7^ PBMCs. This suspension was mixed gently and incubated for another 10 min at 2–8 °C before passing through a sterile magnetic column. The column was then washed with MACS buffer to ensure all purified monocytes were collected in a sterile 15 ml Falcon tube. The flow through purified monocytes were centrifuged at 350×*g* for 5 min at room temperature. Number of viable cells were determined via trypan blue exclusion staining and counted using a haemocytometer and light microscope. Cells at a minimum density of 10^6^ cells/ml were cultured in complete RPMI medium at 37 °C in 5% CO_2_ controlled environment; the procedure was continued for cell surface/intracellular staining as described below or for the transcriptome analysis.

For the transcriptome study, the magnetically purified monocytes were passed through a second step of purification using FACS Aria III based cell sorting. In brief, cells were prepared for sorting, enriched monocyte cell suspensions were incubated with 2 μg/ml Fc human serum immunoglobulin (BD Biosciences; UK) at 4 °C for 10 min to reduce non-specific binding by Fc receptors, and a small volume was aliquoted and labelled as unstained that was used for compensation. The cells were then stained with live/dead (L/D) fixable viability stain (ThermoFisher Scientific, UK) with fluorochrome-conjugated antibodies to CD14 (BioLegend, USA) at a final dilution of 1/100. All experiments were controlled with appropriate isotype antibodies, compensation beads and unstained cells. To verify the purity and quantity of the sorted monocytes, purity check was performed under sterile conditions at 0–4 °C. The purity of the monocytes was 83.3%, 83%, 92.3%, 85.2%, 89.2%, 87%, 90.7%, and 96% respectively for patients RA1–RA8. Representative flow cytometry plots of the sorted monocytes population used in the study are shown in Supplementary Fig. S1.

### Monocyte treatment, cell surface and intracellular cytokines staining

For assessment of intracellular cytokines by flow cytometry, enriched monocytes were seeded in 12-well plates at a minimum density of 10^6^ cells/well in 1 ml of RPMI 1640 complete medium (RPMI 1640 medium containing 10% foetal calf serum (FCS), 100 U/ml penicillin, 100 µg/ml streptomycin and 2 mM l-glutamine) along with 25 and 50 µM PNLA (Cayman Chemicals, USA) or DMSO (Sigma-Aldrich, UK) vehicle control for 24 h and some wells were stimulated with LPS (100 ng/ml) and Brefeldin (PFA; 10 µg/ml) (both from Sigma-Aldrich, UK) for 8–9 h or left un-stimulated as a control and the culturing continued. Following all incubations, the monocytes were collected by detachment of the plastic wells (Supplementary Data S2) and stained for surface fluorochrome-conjugated antibodies specific to monocyte surface receptors CD14, and CD16 (BioLegend, USA) as described in Supplementary Data S2. The monocytes were then permeabilised using 0.3% saponin (ThermoFisher Scientific, UK) diluted in PBS and stained for intracellular cytokines panel TNF-α, IL-1β (BioLegend, USA) and IL-6, IL-8 (BD Biosciences, UK) to quantify the percentage of cytokines producing CD14^**+**^ monocytes as in Supplementary Data S2.

### Flow cytometry analysis

A minimum of 10^6^ cells/mL live cells/sample were collected using forward scatter (FSC) and side scatter (SSC) gating to avoid dead cells and debris and at least 50,000 events were acquired using flow cytometer (BD LSR-FORTESSA) in the 2-3-6-5 configuration (16 colour). Details on acquisition and analysis are provided in Supplementary Data S2.

### Transcriptome analysis

Sorted monocytes were washed with fresh RPMI medium, counted, and seeded in 6-well plates at a concentration of approximately 1 × 10^6^ cells/ml in RPMI 1640 complete medium along with either 25 µM PNLA or DMSO vehicle control for 24 h before stimulation with LPS (100 ng/ml) or vehicle control for 4 h. The cell suspensions were then collected and washed with Ca^2+^ or Mg^2+^ free Dulbecco's PBS medium ( ThermoFisher Scientific, UK). The adherent cells were detached from the plastic by addition of 1 ml accuatase; cell dissociation reagent (ThermoFisher Scientific, UK) pH 6.8/well, incubated for 5–8 min at 37 °C with 5% CO_2_, and then washed. The cell suspension was centrifuged at 400×*g* at room temperature for 5 min and kept at − 80 °C in 350 µl buffer RLT (provided in RNeasy Mini kit) supplemented with 10% of 2-mercaptoethanol (Gibco Life Science, UK) as a lysate till RNA extraction.

### RNA extraction

Total RNA was isolated using a RNeasy mini kit (Qiagen, Hilden, Germany) from purified monocytes following 100 ng/ml LPS and 25 µM PNLA or vehicle treatment. RNA was purified using a RNeasy kit on-column with DNase I digestion (Qiagen) as described in Supplementary Data S3. The cell lysates were stored at − 80 °C and then passed through a series of spin columns to first bind genomic DNA, then RNA and finally to elute high-quality RNA using the methodology by Takala et al. in 2021^6^. A quality control check for RNA was assessed as described in Supplementary Data S3.

### Library construction, sequencing, and data processing

#### Library preparation including ribosomal RNA depletion

In collaboration with the Wales Gene Park (Cardiff University), 5 ng of total RNA was depleted of ribosomal RNA using the NEBNext rRNA Depletion Kit (Human/Mouse/Rat), (New England BioLabs, UK Ltd). Ribosomal RNA depletion of each sample was assessed using the Agilent 4200 TapeStation with hsRNA ScreenTape (Agilent Technologies, Inc, UK).

The sequencing libraries were prepared using the NEB Ultra II Directional RNA Library Prep Kit for Illumina (New England BioLabs, UK Ltd) protocol. The steps included RNA fragmentation and priming, 1st strand cDNA synthesis, 2nd strand cDNA synthesis, adenylation of 3′ ends, adapter ligation (adapter diluted 1:199) and PCR amplification (16-cycles). Following the addition of the PCR enrichment master mix, a unique index primer was added to each sample. The standard fragmentation procedure of 15 min at 94 °C for samples for intact RNA (> 7) was reduced to 8 min at 94 °C for partially degraded RNA with RIN values 2–6. Except for the replacement of RNAClean XP beads and SPRI select beads by AMPure XP beads (Beckman Coulter) the manufacturer’s instructions were followed. The libraries were validated using Agilent 4200 TapeStation and high sensitivity DNA ScreenTapes (Agilent Technologies, Inc, UK) to ascertain the insert size, and the Qubit (ThermoFisher Technologies, UK) was used to perform the fluorometric quantitation.

#### RNA-sequencing (RNA-seq)

Following library quantification and validation, the normalized library pool was sequenced on one lane of an S1 (200 cycle) flow cell using XP workflow and 2 × 100 bp paired end dual indexed format on the NovaSeq6000 sequencing system (Illumina Inc, USA) according to the manufacturer’s instructions. To conform to ENCODE guidelines, libraries were sequenced to have > 54 million mapped reads (encodeproject.org/documents/cede0cbe-d324-4ce7-ace4-f0c3eddf5972).

#### RNA-seq data processing and read mapping strategy

Paired end reads from the Illumina sequencing were trimmed with Trim Galore^8^ and assessed for quality using FastQC^9^, using default parameters. Reads were mapped to the human GRCh38 reference genome using STAR software^10^ and reads per gene were assigned using the featureCounts software^11^ with the GRCh38.96 gene build gene transfer format (GTF). Both the reference genome and GTF were downloaded from the Ensembl FTP site^12^. Duplicate reads were identified and marked using MarkDuplicates in Picard (Broad Institute).

#### Normalisation and identification of differentially expressed genes (DEGs)

Differential gene expression analyses used the DESeq2 package^13^. Genes were discarded from the analysis if differential expression failed to be significant (p value < 0.05)^13^. The initial WGT data were assessed with the web-based tool Morpheus (https://software.broadinstitute.org/morpheus/) using heatmaps to view changes in gene expression. The data were then separated into 3 groups; vehicle treated, vehicle treated/LPS stimulated, and PNLA treated/LPS stimulated monocytes for functional analysis including gene set enrichment analysis, gene ontology and further downstream pathway analysis.

### Heatmap of DEGs and principal component analysis (PCA)

A heatmap and PCA are shown in Supplementary Figs. S2, S3. The heatmap was generated using broad Morpheus software and visualizations used fragments per kilobase per million mapped fragments (FPKM) reads and log2 fold change (log2 FC) comparing PNLA-treated and LPS-stimulated monocytes with those treated with vehicle and LPS from RA patients at equivalent time point (Supplementary Fig. S2). Data sets were hierarchically clustered using 1 minus Pearson’s correlation coefficient. PCA was performed in R using normalized data from the DESeq2 analysis, and data were clustered using the top 50 most DEGs over all comparisons (Supplementary Fig. S3). Each plot shows the results of the first two principal components.

### Statistical analysis

The statistics used were dependant on the experiments performed and depending on data distribution. Normality of data was tested using the Shapiro–Wilk test and confirmed with histograms and Q–Q plots in SPSS Statistics (version 23; IBM Corp., Armonk, NY, USA). FCS data files were analysed using FlowJo (version10.7, BD, UK) and the NovoExpress flow cytometry software (version 1.5.6). Percentage of cytokine expressing CD14^+^ monocytes was calculated using Microsoft Excel (version 16.52, Microsoft 365 Subscription, UK). Graphs and statistical analysis were performed using Graph Pad Prism (version 8 GraphPad Software, La Jolla, CA, USA). One way ANOVA was used to compare multiple groups followed by post hoc analysis for pairwise comparisons. Spearman correlation was used to analyse the linear association using SPSS statistics (version 26; IBM Corp, Armonk, NY, USA) and 2 tailed Mann Whitney U test was applied to find the comparison between 2 groups. All values are presented as mean ± SEM, p value < 0.05 was considered significant for all the analysis.

### Ethics approval and consent to participate

The study was approved by the Research Ethics Committee for Wales 3 (reference no. 12/WA/0045) University Hospital of Wales. All patients signed a consent form approved by the local research and governance boards (University Hospital of Wales) before participating in this study.

### Consent for publication

Yes.

## Results

### The relative intracellular cytokines levels expressed by monocytes were significantly reduced by PNLA

The analysis was carried out on the enriched monocytes that constitute about 85–90% of all circulating blood monocytes, which we represent and refer to as CD14^**+**^ monocytes.

**Figure 1:**
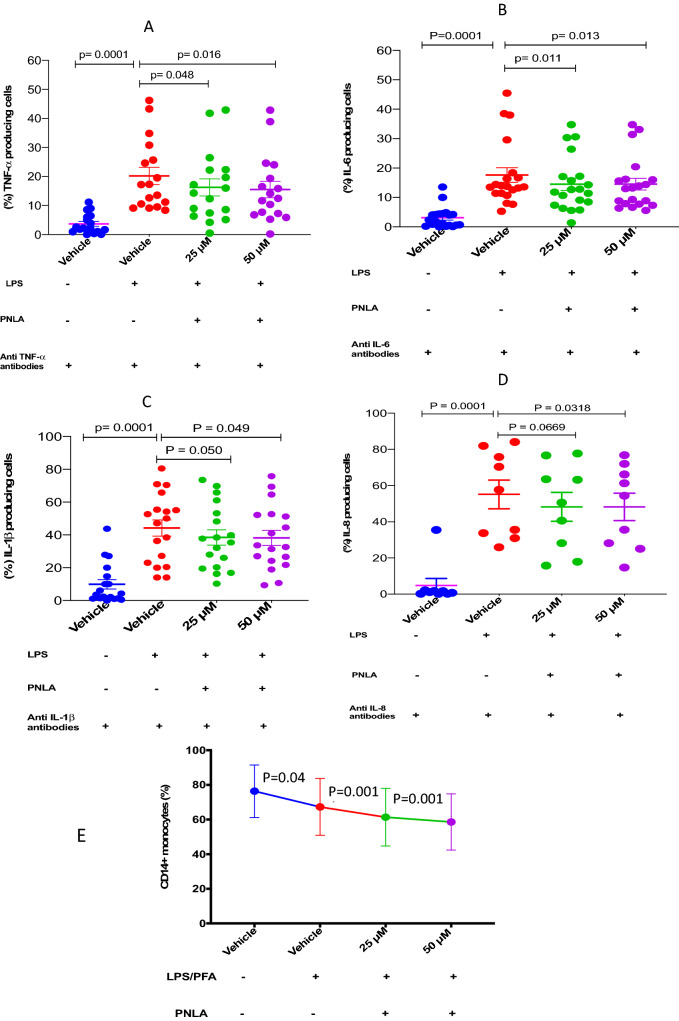
CD14^+^ monocytes expressing inflammatory cytokines were reduced significantly following PNLA treatment. TNF-α, IL-6 IL-1β, and IL-8 were reduced (**A**–**D**) respectively and PNLA at 25 or 50 µM reduced the percentage of monocytes (**E**). Purified monocytes were incubated with 25 or 50 µM PNLA for 24-h followed by 100 ng/ml LPS stimulation for 9 h. Then, cells were stained and analysed for the expression of TNF-α, IL-6, IL-1β and IL-8 antibodies using flow cytometry; all samples run in duplicates or triplicates. The data are presented as mean ± SEM, each dot (•) represents the average of one participant. Statistical analysis was performed using one-way ANOVA and a  Dunnett's post-hoc-test. TNF-α, IL-6, IL-1β were assessed in (n = 20), and IL-8 in (n = 9) RA patients.

LPS stimulation increased CD14^**+**^ monocytes producing TNF-α (20.6 ± 2.96%) (p < 0.0001) in comparison with non-stimulated monocytes (3.69 ± 0.83%). Pre-incubating monocytes with 25 or 50 µM PNLA reduced the percentages of CD14^**+**^ cells producing TNF-α to 16.1 ± 2.96% and 15.2 ± 2.81% respectively (p = 0.048 and 0.016 respectively) (Fig. 1A).

Percentage of CD14^+^ monocytes expressing IL-6 increased from 3.11 ± 0.78% in non-stimulated cells to 17.9 ± 2.49% (p < 0.0001) after LPS stimulation. Pre-incubating monocytes with 25 or 50 µM PNLA reduced the percentages of CD14^**+**^ cells expressing IL-6 to 14.2 ± 2.07% and 14.2 ± 2.00% respectively (p = 0.011 and 0.013 respectively) (Fig. 1B).

LPS stimulation increased CD14^**+**^ monocytes producing IL-1β to 42.99 ± 4.94 (p < 0.0001) in comparison with non-stimulated monocytes (9.86 ± 2.91%). Pre-incubating monocytes with 25 or 50 µM PNLA reduced the percentages of CD14^**+**^ cells expressing IL-1β to 37.1 ± 3.62% and 37.0 ± 3.61% respectively (p = 0.050 and 0.049 respectively) (Fig. 1C).

LPS stimulation increased percentage of CD14^+^ monocytes expressing IL-8 to 55.9 ± 7.91% (p < 0.001) in comparison with 4.81 ± 3.8% for non-stimulated monocytes. Pre-incubating monocytes with 25 or 50 µM PNLA slightly reduced the percentages of CD14^+^ cells expressing IL-8 to 47.2 ± 7.97% and 48.2 ± 6.58% respectively (p = 0.0669 and 0.0318 respectively) (Fig. 1D). The differences were statistically significant only for 50 µM PNLA.

### CD14^+^ monocyte percentages were reduced following PNLA treatment

Figure 1E shows the percentage of CD14^+^ monocytes, which was significantly reduced following PNLA treatment. Vehicle versus LPS stimulated monocytes was 76.37 ± 3.45 (p = 0.04), while PNLA treated versus LPS stimulation were 61.37 ± 5.92% and 58.59 ± 8.70% for 25 and 50 µM PNLA respectively (p = 0.001). NovoExpress flow cytometry software was used to determine the gates and the gating strategy.

### Intracellular cytokines expression upon PNLA treatment did not significantly correlate with patients’ demographic, clinical, and laboratory markers

There was no statistically significant difference/correlation between percentage of reduction in CD14^+^ monocytes expressing TNF-α, IL-6 or IL-1β cytokines by PNLA against a variety of clinical indices and disease biomarkers after adjustment for multiple comparisons (Supplementary Table S3).

### Heatmap of DEGs and PCA

A heatmap (Supplementary Fig. S2) showed the main clusters of DEGs of all treatment conditions mentioned. It can be visualised as two main upper and lower clusters. In the left upper cluster, the expression of vehicle treated monocyte genes were upregulated while PNLA and LPS treatment downregulated those genes on the right side. Conversely, in the lower cluster, the opposite pattern can be visualised. PCA results (Supplementary Fig. S3) also show samples clustering within groups demonstrating that inter-sample variation in gene expression is all within the normal range and not greater than the biology we hope to observe.

### Graphical summary and canonical pathways

**Figure 2:**
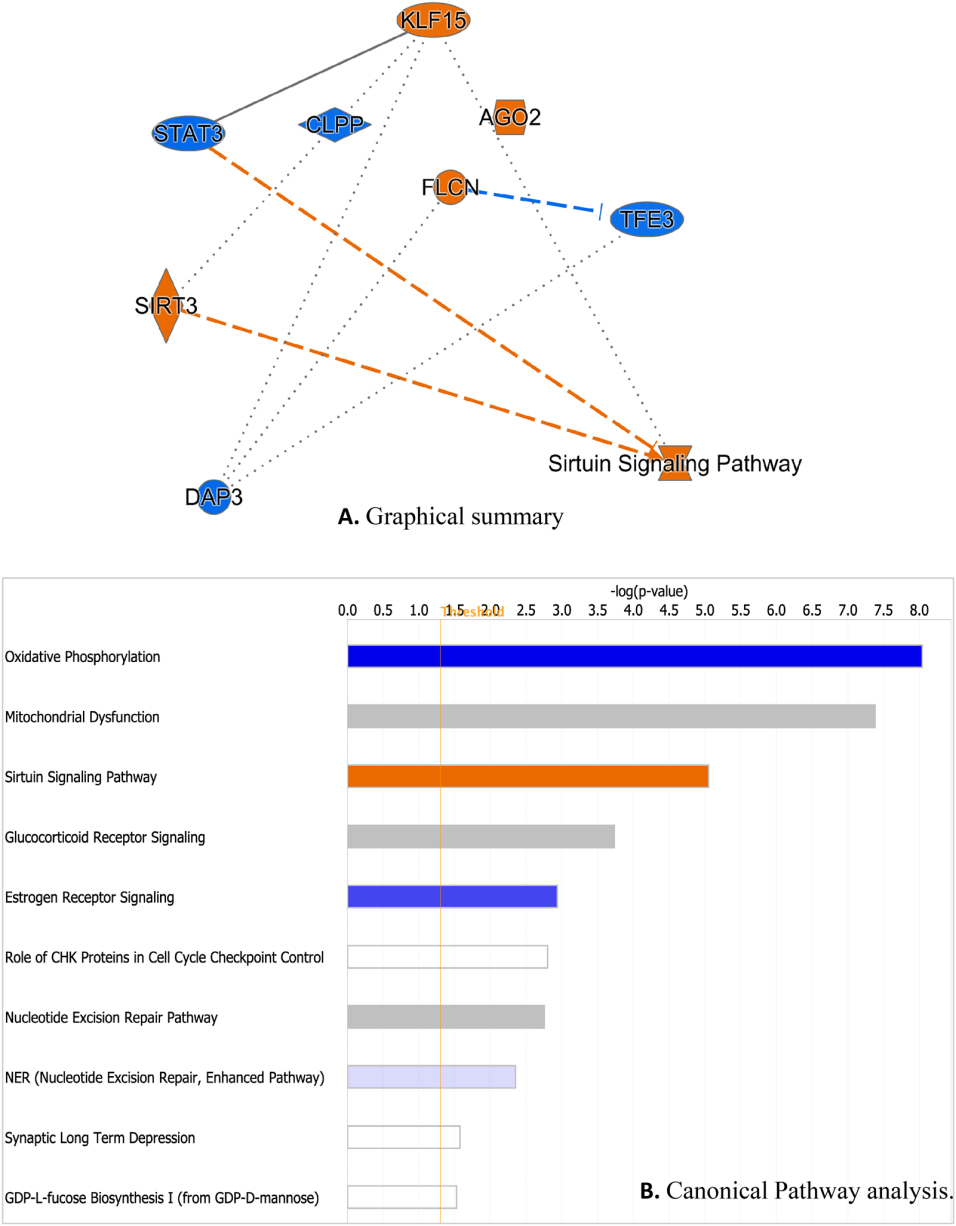
Graphical summary and canonical pathway analysis. (**A**) Graphical summary showing the most significant transcription factors and regulators affected by PNLA. The orange colour goes with the direction of activation while the blue is the inhibition state. (**B**) Canonical pathways bar chart for PNLA effect on activated monocytes. The top 10 most significant pathways are shown, as calculated by Fisher’s exact test right tailed. The colour of the bar is a measure of z-score which reflects the direction of the expression change (pathway activation or inhibition). An orange bar represents an overall positive z-score (majority of genes are upregulated) while a blue bar represents an overall negative z-score (majority of genes are downregulated). White bars possess a z-score which is zero or close to zero. Grey bars highlight pathways for which IPA is unable to make a prediction. The orange line graph represents the ratio (the number of genes that meet cut-off criteria in the dataset, divided by the total number of known genes attributed to that pathway in the IPA reference gene set).

Data were analysed through the use of IPA (QIAGEN Inc., https://www.qiagenbioinformatics.com/products/ingenuity-pathway-analysis). Graphical summary provides an overview of major biological themes in IPA (version: 33559992, Qiagen, USA); it selects the most significant entities in the core analysis and shows how they relate to each other. Biological process and regulators included have z-score ≥ 2. Nodes are coloured by their activity predicted in the analysis where orange nodes are predicted to be activated with z-score ≥ 2. In contrast, blue coloured entities with z scores ≤ 2 are predicted to be inhibited. As summary, PNLA treatment activated KLF15, AGO2, FLCN, SIRT3 and Sirtuin signalling pathways and inhibited STAT3, TFE3, CLPP and DAP3 (Fig. 2A).

IPA was used to identify canonical pathways associated with differentially regulated transcripts identified between genotypes affected by PNLA and relevant pathways. They were determined by their p value (Fig. 2B). The canonical pathway most significantly associated with the differentially expressed transcripts was in ‘oxidative phosphorylation’ (p value = 9.14E−09) (Supplementary Fig. S4). Of the 91 genes attributed to this pathway in the IPA reference dataset, 13 were differentially expressed in the experimental dataset which are ATP5PF, COX15, COX17, MT-ATP6, MT-CO2, MT-ND1, MTND2, MTND4, MTND5, MT-ND4L, NDUFA7, NDUFA8, and NDUFB4. These proteins are all located in the cytoplasm of the inner mitochondrial membrane and function as enzymes or transporters for the mitochondria. Although oxidative phosphorylation is a vital part of metabolism, it produces reactive oxygen species (ROS) such as superoxide (O2-) and hydrogen peroxide (H_2_O_2_), which leads to propagation of free radicals, damaging cells and contributing to immune alterations and disease. Sirtuin signalling pathway was the third most significant canonical pathway that was activated in response to PNLA treatment versus LPS stimulated (p = 8.89E−06) after mitochondrial dysfunction (p = 4.18E−08).

### Comparison analysis and upstream regulators

**Figure 3:**
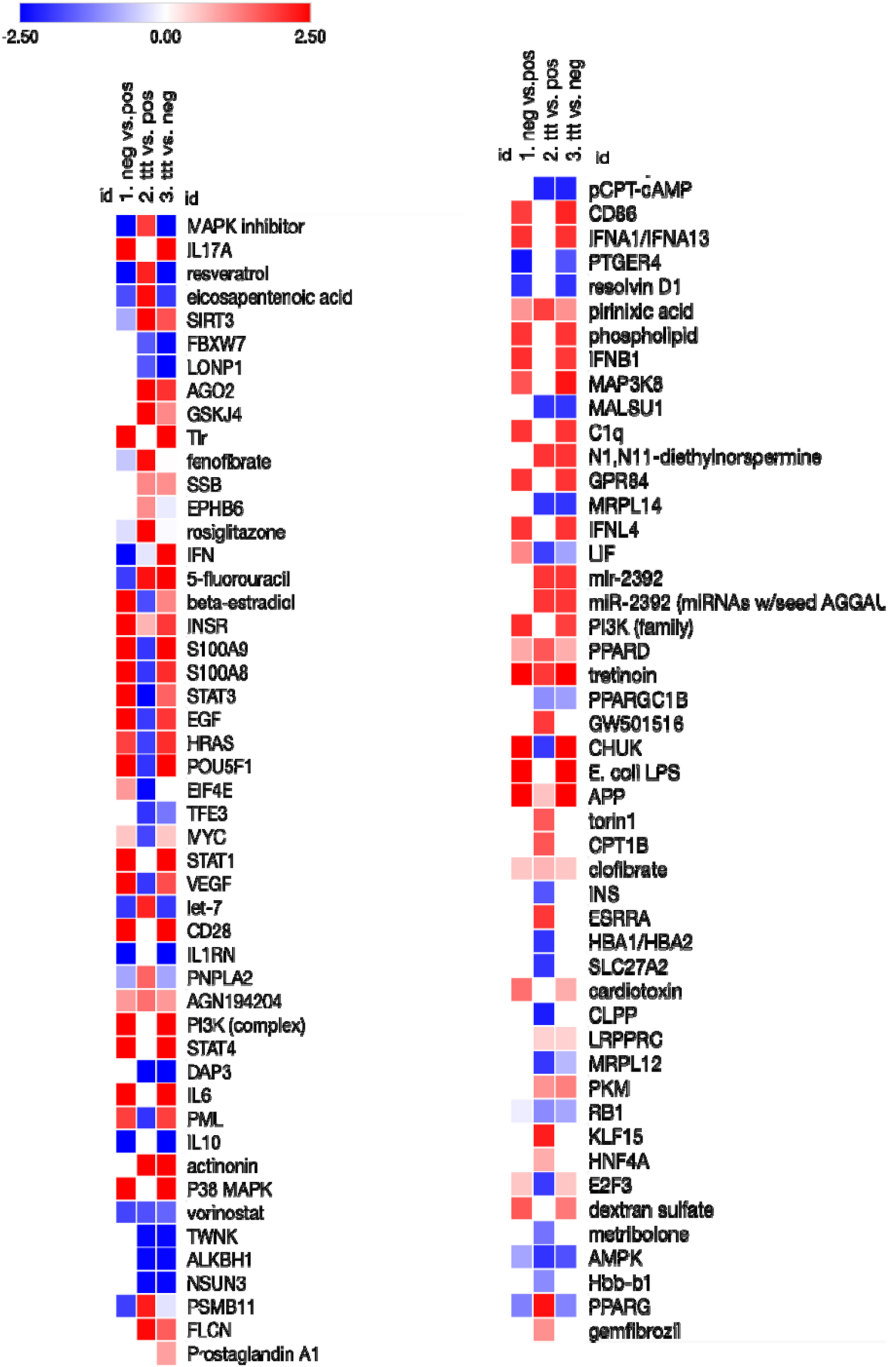
Heatmap of differentially expressed upstream regulators for comparison of RA patients. Data were analyzed through the use of IPA (Qiagen  Inc., https://www.qiagenbioinformatics.com/products/ingenuity-pathway-analysis). IPA analysis of DEGs associated with upstream regulators that predicted activated state (red) and predicted inhibited state (blue) are shown. Data files were exported for those genes from IPA and reproduced heatmaps using Broad Morpheus software available at (https://software.broadinstitute.org/morpheus/). Relative expression heat maps of the differentially expressed regulators regulated by; (1) unstimulated (vehicle) vs LPS-stimulated monocytes (2) PNLA-treated LPS-stimulated vs vehicle-treated LPS-stimulated monocytes, and (3) PNLA-treated LPS-stimulated vs unstimulated (vehicle-treated) monocytes (Log2 FC > 1.2, p < 0.05) are shown. The regulators shown here are mainly involved in cytokine production, lipid and fatty acid metabolism, drugs used for treating hyperlipidaemia, miRNAs and transcription factors. *LPS* Lipopolysaccharide, *PNLA* pinolenic acid, *ttt* treatment.

Using IPA software, the comparison analysis was performed for all experimental conditions; vehicle, LPS stimulated, and LPS stimulated/PNLA treated monocytes. In this analysis, we particularly sought at upstream analysis for the genes involved in the transcription of inflammatory cytokines, lipid metabolism and fatty acids actions, some drugs used for treating hyperlipidaemia and miRNAs. Data files were further exported for those genes from IPA and reproduced heatmaps using Broad Morpheus software. Heatmap visualisations using log2FC > 1.2 are shown in Fig. 3. Top predicted upstream regulators affected by PNLA treatment are shown in Supplementary Table S4, Supplementary Data S4 and Supplementary Fig. S5.

### miRNAs impacted by LPS and PNLA

**Figure 4:**
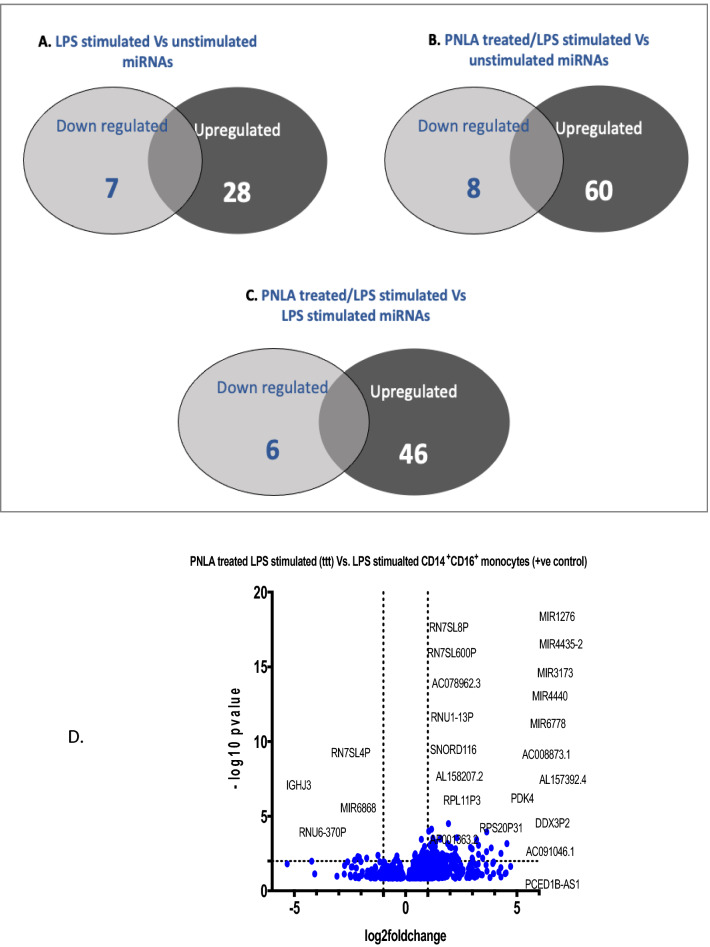
Venn diagrams showing the significantly affected miRNAs; (**A**) unstimulated (vehicle) vs LPS-stimulated monocytes (**B**) PNLA-treated LPS-stimulated vs vehicle-treated LPS-stimulated monocytes, (**C**) PNLA-treated LPS-stimulated vs unstimulated (vehicle-treated) monocytes (p value < 0.05), and (**D**) Volcano plots of global significant and non-significant genes of protein coding and non-coding DEGs of PNLA treated/LPS stimulated versus LPS stimulated monocytes.

Venn diagrams (Fig. 4) showed that 35 miRNAs were differentially expressed in LPS stimulated versus unstimulated monocytes (7 were downregulated and 28 upregulated) (Fig. 4A). PNLA treated/LPS stimulated versus unstimulated monocytes identified 68 miRNAs that were differentially expressed with 8 downregulated and 60 upregulated (Fig. 4B). PNLA treatment/LPS stimulated versus LPS stimulated monocytes showed 6 downregulated miRNAs and 46 upregulated miRNAs (Fig. 4C). List of these miRNAs are provided in Supplementary Table S5A,B. (Fig. 4D) show volcano plots of protein coding/noncoding genes of PNLA treated and LPS stimulated versus LPS stimulated monocytes.

### miRNAs target mRNAs

We determined mRNA targets of miRNA and the pathways involved in inflammation and lipid/cell metabolism by specific miRNA target prediction filter in IPA. This selected miRNAs–mRNA with a confidence of high or moderate prediction based on database.

Using miRNA target filter, top 72 significantly regulated miRNAs affected by PNLA treatment and LPS stimulations were uploaded into the core analysis, with 39 miRNAs having target information available. The associated mRNAs and pathways related are shown in Table 1. Expression fold change and expression p value were determined based on calculated Fisher’s exact test right tailed in IPA as shown.

**Table 1:** Selected miRNAs target mRNA and the relevant associated pathways as per IPA database.

miRNA ID	Expr p value	Expr log ratio	mRNA symbol	Expr p value	Expr log ratio	Pathway
miR-4521	0.0542	2.853	ACAA2	0.0034	0.91	Fatty acid β-oxidation I, Glutaryl-CoA degradation, I, ketogenesis, ketolysis, super pathway of cholesterol biosynthesis
miR-7150	0.0429	1.805				
miR-8066	0.0423	3.642				
miR-4440	0.0136	2.997	ATMIN	0.0304	− 0.243	Role of CHK Proteins in cell cycle checkpoint control
miR-3173	0.0011	1.975	CRABP2	0.0407	1.488	Acute phase response, retinoid acid mediated apoptosis signalling
miR-374B	0.0034	1.382	ETFA	0.0498	0.248	NAD signalling pathway
miR-1909	0.0303	− 0.548	FZD2	0.0334	− 1.057	Adipogenesis pathway, osteoarthritis pathway, role of macrophages, fibroblasts and endothelial cells in rheumatoid arthritis, role of osteoblasts, osteoclasts and chondrocytes in rheumatoid arthritis
miR-4440	0.0136	2.997	GHRL	0.0388	0.429	Leptin signalling in obesity
miR-548L	0.0712	1.677				
miR1260B	0.0206	0.901	JMJD7-PLA2G4B	0.05	0.483	ERK/MAPK signalling, glucocorticoid receptor signalling, MIF regulation of innate immunity, MIF-mediated glucocorticoid regulation, pathway, p38 MAPK signalling, phospholipase C signalling, VEGF family ligand-receptor interactions, CCR3 signalling in eosinophils
miR-646	0.0702	3.95				
miR-2861	0.0611	− 1.178	LSM1	0.0312	− 0.282	Systemic lupus erythematosus signalling
miR-3934	0.0758	1.072	MT-ATP6	0.0307	− 0.991	Mitochondrial dysfunction, oxidative phosphorylation, sirtuin signalling pathway, estrogen receptor signalling, glucocorticoid receptor signalling
miR-671	0.0386	− 0.35				
miR-3188	0.0358	1.958				
miR-7150	0.0429	1.805	PAIP1	0.0265	− 0.209	CSDE1 signalling pathway, EIF2 signalling, Insulin secretion signalling pathway
miR-3173	0.0611	1.975	PDK4	0.000364	3.19	Glucocorticoid receptor signalling. Reelin signalling, senescence pathway
miR-2861	0.0611	− 1.178				
miR-626	0.0446	1.486				
miR-28	0.0429	2.181				
miR-7150	0.0358	1.805				
miR-3188	0.0470	1.958				
miR-3934	0.0611	1.072				
miR-1909	0.0303	− 0.548	SIGIRR	0.0445	1.124	NF-κB Signalling, TLR signalling, TREM1 signalling
miR-7150	0.0429	1.805				
miR-6868-5P	0.0025	1.501				
miR-637	0.0063	− 1.747	SPCS1	0.00457	− 0.389	Insulin secretion signalling pathway

**Figure 5:**
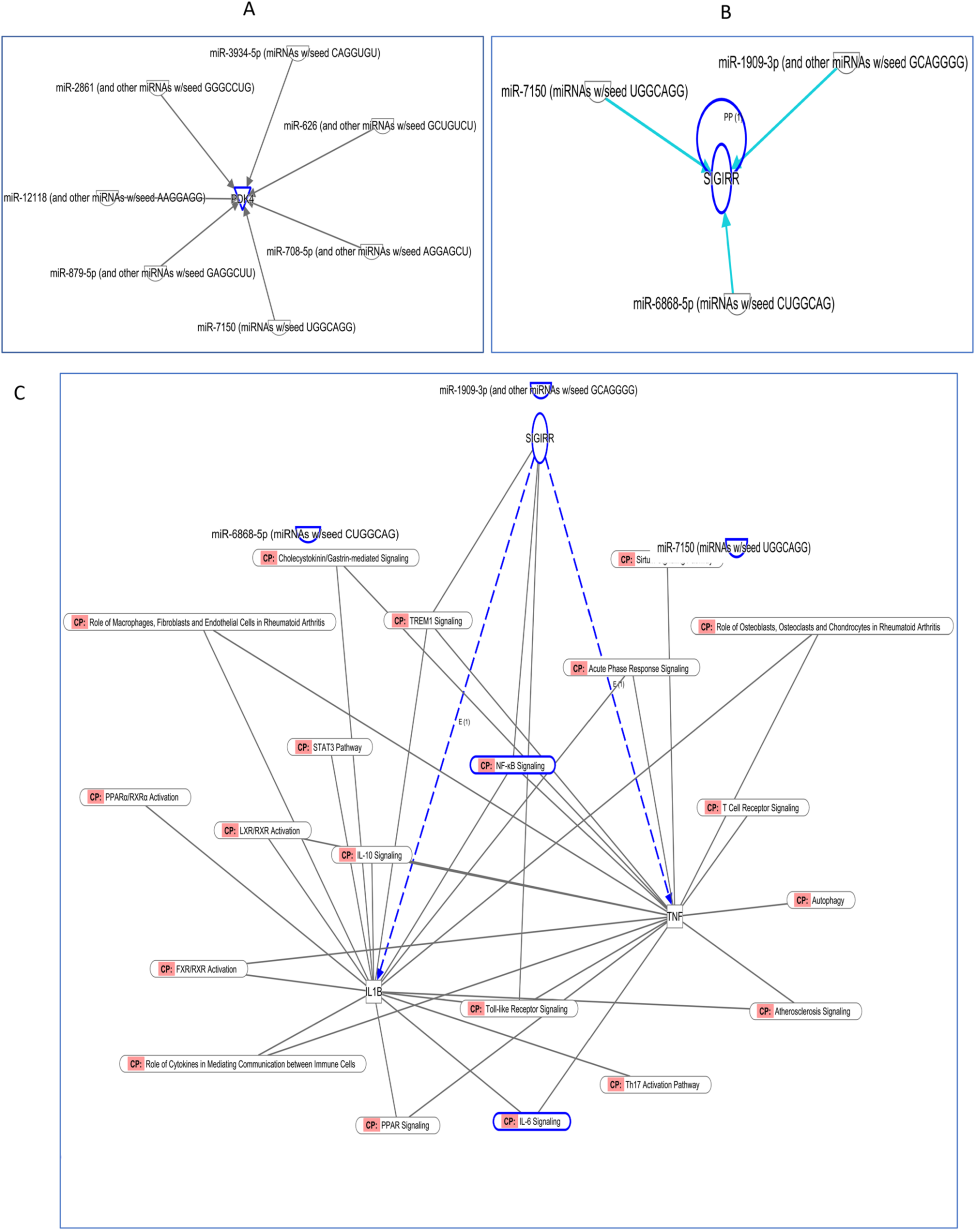
miRNAs targeted PDK4 and SIGIRR is downstream for miRNAs and upstream of metabolic and inflammatory pathways. (**A**) PDK4 mRNA is a target for miR-3173, miR-2861, miR-626, miR-28, miR-7150, miR-3188, miR-3934, and miR-1909. (**B**) SIGIRR mRNA is a target for miR-1909-3P, miR-7150, miR-6868-5P and (other miRNAs  with seed GCAGGGG) targeted SIGIRR mRNA. (**C**) SIGIRR upstream for many signalling pathways involved in regulation of transcription factors, inflammatory cytokines, and metabolic molecules. It negatively regulates NF-κB Signalling, TLR signalling, and TREM1 signalling. *CP* Canonical pathway.

Interestingly, one of mRNA targeted is PDK4, (Table 1) which is targeted by several miRNAs: miR-3173, miR-2861, miR-626, miR-28, miR-7150, miR-3188, miR-879-5p, miR-393-5p, miR-708-5p and miR-12118 (Fig. 5A). This is consistent with the previous observation in PNLA treated PBMC^6^.

SIGIRR is a mRNA that was actively targeted by miRNAs upon PNLA treatment. It negatively modulates immune responses because of its role in inhibition of NF-κB and TLRs signalling. SIGIRR is mostly expressed intracellularly in primary human monocytes, macrophages, and dendritic cells (DCs)^14^ SIGIRR is targeted by 3 miRNAs as shown in Fig. 5B, which are miR-7150, miR-1909-3p and miR-6868-5p based on IPA database. SIGIRR downstream regulatory effects are shown in Fig. 5C. MT-ATP6 is targeted by miR-3934, miR-671, and miR-3188; bioinformatic analyses also showed that the targets of these miRNAs are “metabolic and lipid regulating mRNAs” such as JMJD7-PLA2G4B, ACAA2, ETFA, SPCS1 and GHRL, which are involved in metabolic signalling pathways, including, glucose metabolism, cholecystokinin (CCK) secretion, mitochondrial ATP synthase, NAD signalling pathway, oxidative phosphorylation, and fatty acid β-oxidation as shown in Table 1.

## Discussion

We aimed to investigate the effect of PNLA on activated monocytes from patients with RA. The data obtained from this study suggest a direct link between PNLA supplementation and modulation of activated monocytes from patients with RA. These effects have the potential to express anti-atherogenic, anti-inflammatory mRNAs and  miRNAs and reduce the expression of intracellular inflammatory cytokines TNF-α, IL-6, IL-β, and IL-18. In addition, PNLA reduces the percentage of LPS activated monocytes invitro (Supplementary Data S5).

Many DEGs from monocyte transcriptome, amongst these PDK4, ACADVL, CPT1A and SLC25A20 (Supplementary Table S6A), were also identified previously in PBMCs from RA patients treated with PNLA^6^. However, new transcription factors and biologic pathways were identified amongst these SIRTs were activated by PNLA. SIRTs signalling pathways were suggested as therapeutic targets in RA and atherosclerosis^15,16^. SIRT1 was found to be upregulated in synovial tissues and cells from patients with RA compared to OA^17^. SIRT1, a NAD^**+**^ dependent histone deacetylase, down regulates both the innate and adaptive immune response in mice. SIRT1 can inhibit NF‐κB pathway directly by itself or indirectly via forkhead family of transcription factors (FoxO)^16,18^. SIRT1 deacetylase was initially shown to deacetylate RelA/p65 at lysine 310 residue, which leads to reduced NF-κB transcriptional activity^19–21^. Substrates of SIRT1 are particularly abundant and include NF-κB RelA/p65, AP-1 family transcription factor c-Jun, and c-Myc. It was also reported that SIRT1 suppressed IL-12 production in human DCs through a direct interaction with the NF-κB transcription factor c-Rel^15^. This can regulate NF-κB, which regulates the expression of cytokines, chemokines, and other pro-inflammatory agents. Also, recent studies suggest that SIRT1 has functions in chondrocytes and synoviocytes during inflammatory arthritis and modulates a variety of cell types during arthritis^7,20,22,23^. Overexpression of an enzymatically inactive form of SIRT1 reduced LPS-induced levels of TNFα in monocytes^22^.

SIRTs have the potential to reduce the inflammatory component of atherosclerosis and may have the potential to alter the course of atherogenesis^24^ (Supplementary Fig. S6). SIRT1 is highly expressed in human vascular endothelial cells (ECs) and regulates many cellular processes essential for cell survival, apoptosis, inflammation, stress resistance, cell growth, cell senescence and metabolism^24^. SIRT1 deficiency contributes to increased inflammation, oxidative stress, foam cell formation, impaired NO production and autophagy, thereby promoting vascular atherosclerosis and aging^18,25,26^.

SIRT3 regulates several mitochondrial functions and has important roles in maintaining homeostasis. SIRT3 is believed to be a positive regulator of macroautophagy in adipocytes. In mature adipocytes, overexpression of SIRT3 activated macroautophagy, mainly on lipid droplets (LDs), through activating the AMP-activated protein kinase-Unc-51 like autophagy activating kinase 1 pathway, which in turn results in smaller LD size and reduced lipid accumulation. Moreover, SIRT3 overexpression causes instability of LDs in adipocytes and participates in the control of FA metabolism. SIRT3 knockout mice demonstrate abnormal lipid metabolism associated with abnormal accumulation of triglycerides in the livers of these animals during fasting^24^.

STAT3 and TFE3 are transcription factors that were inhibited by PNLA. STAT3 is an established target in RA as inhibitors of JAK and IL-6 are approved treatment. TFE3 and TFEB are transcriptional regulators important in the activation of macrophages^27^. Mice deficient of either TFEB or TFE3 showed reduced expression of cytokines. TFEB and TFE3 are master regulators of macroautophagy/autophagy and lysosome function in activated macrophages, and raises the possibility that these transcription factors may be of central importance in linking autophagy and lysosome dysfunction with inflammatory disorders, and the transcription of pro-inflammatory cytokines such as TNF-α and IL-1β requires the presence of both TFEB and TFE3^28^.

DAP3 was found to be the topmost significant upstream regulator affected, is a mitochondrial ribosomal small subunit protein that is involved in mitochondrial physiology, apoptosis and TNF associated cell death pathways^29^. Overexpression of DAP3 leads to a significant increase in cell death and osteoclast formation. DAP3 appears to act downstream of pro-apoptotic stimuli such as IFN-γ, TNF-α and Fas ligand, and is upstream to several caspases, such as caspase 8 and 9^29^.

To the authors’ best knowledge, this is also the first study to show that many miRNAs targeted mRNA were modulated by PNLA. Amongst these are, PDK4, SIGIRR and MT-ATP6. PDK4 regulates TNF-α and NF-κB^6,30^ whereas SIGIRR downstream regulatory effects include IL-1β, TNF-α, NF-κB and TRIM1 signalling pathways. SIGIRR was first characterised as an inhibitor of IL-1R and TLR signalling by interaction with IRAK-1, and TRAF-6 in 293 human cell line^14^. SIGIRR overexpression inhibited TLR-induced TNF-α, IL-10 and IFN-γ-inducible protein 10 (IP-10) production in HMDMs and DCs. The role of SIGIRR as an inhibitor of inflammation was confirmed in vivo, since SIGIRR (−/−) mice developed a more severe disease in CIA models^14^. Overexpression of SIGIRR in primary human DCs as well as in M-CSF differentiated macrophages resulted in the inhibition of TLR-2/6, TLR-3, TLR-4, TLR-5, TLR-7/8, and IL-1R signalling^14^. Overexpression of SIGIRR in human RA synovial cells led to the inhibition of spontaneously produced cytokines ^14^. MT-ATP6 is essential for mitochondrial function, oxidative phosphorylation, sirtuin signalling, and glucocorticoid receptor signalling (Table 1).

AGO2 (Argonaute 2) encodes a member of the Argonaute family of proteins which play a role in RNA interference. The encoded protein is highly basic, silencing of miRNA is mediated by Ago proteins.

let7 is a family of miRNAs that has variety of anti-inflammatory and anti-atherogenic actions^31–33^. All members of the let-7 family have been linked to regulation of vascular EC and SMCs, which are critical in the pathogenesis of atherosclerosis^32,33^. Let7 negative feedback regulation was involved in VSMC proliferation and migration^32^.

KLF15 is a member of the KLFs family, which are transcription factors important in regulating inflammation and involved in numerous pathological processes associated with atherogenesis and associated with regulation of various signal transduction pathways^34^. Mice with systemic and smooth muscle–specific deficiency of KLF15 exhibited an aggressive inflammatory vasculopathy^35^. KLF15 deficiency is seen in several CV disorders, such as heart failure and aortic aneurysm^34^. KLF15 expression is reduced in atherosclerotic tissues from human aortic samples with an approximately sevenfold reduction in KLF15 mRNA expression compared with nonatherosclerotic control aortae^34^. It is an essential regulator of VSMC proinflammatory activation, which alters the acetylation status and activity of the proinflammatory factor NF-κB^35^.

Overexpression of KLF15 in human EC line (Eahy926) exhibited a protective effect against TNF-α induced dysfunction^34^. Overexpression of KLF15 markedly suppressed the rate of cellular adhesion, and downregulated levels of MCP-1, ICAM-1, TGF-β and phospho-p65 (p-p65) in TNF-α induced Eahy926 cells. Overexpression of KLF15 markedly enhanced cell viability and did not exhibit a significant difference regarding the quantity of released nitric oxide (NO) compared with the control group in same cell model^34^.

For the transcriptome study, in order to minimise heterogenicity, we have recruited patients on intravenous biologic treatments. Blood samples were drawn before intravenous treatment as part of clinical care. Therefore, serum monoclonal antibodies were at the trough levels before each cycle of treatment, in particular, rituximab has a half-life of 3 weeks, and it is given 6 monthly for the treatment of RA. It is therefore unlikely for rituximab to be present in the blood sample. Furthermore, rituximab is an anti-CD20 monoclonal antibody, which acts by depleting B cells and has no direct effect on monocytes. Therefore, rituximab should not affect the transcriptome of monocytes.

## Limitations and future work

Results of mRNA and miRNA from transcriptomic study are exploratory and need to be confirmed by qPCR and/or western blot at protein level. In addition, the repressive effect of distinct miRNA species on their putative targets needs to be validated and confirmed by luciferase assays. RA is a heterogenous disease even though we did not find any association between disease features and the effect of PNLA on cytokine expression by monocytes, we cannot adequately address whether the transcriptome profiles may be influenced by clinical features relative to this sample size. The anti-inflammatory effect of PNLA on LPS stimulated CD14 monocytes will need to be confirmed by prospective randomised control trial along with the measurement of effectiveness at the protein level.

## Conclusion

PNLA reduces the proportion of activated CD14 monocytes expressing TNFα, IL-6, IL-1β and IL-8 in active RA patients. PNLA regulates several miRNAs that target key mRNA involved in modulation of the inflammatory, metabolic/lipid and mitochondrial pathways. Although replication and validation are needed, results in this study are promising, and when considered in conjunction with previously demonstrated immunomodulatory effects of PNLA.

Dietary supplements of PNLA may be beneficial for articular and vascular disease in patients with RA and atherosclerosis. Although both diseases pathogenesis is intercorrelated, there is no current guidelines that are addressing the direct link for treating RA patients and CVD, and this may highlight the promising benefits of this PUFA as a potential therapeutic agent.

## Supplementary Information

Supplementary Information 1.

Supplementary Information 2.

Supplementary Figure 1.

Supplementary Figure 2.

Supplementary Figure 3.

Supplementary Figure 4.

Supplementary Figure 5.

Supplementary Figure 6.

Supplementary Tables.

## Data Availability

The datasets generated and/or analysed during the current study are available in the ArrayExpress repository, link: https://eur03.safelinks.protection.outlook.com/?url=https%3A%2F%2Fwww.ebi.ac.uk%2Farrayexpress%2Fexperiments%2FE-MTAB-11585&data=04%7C01%7Ctakalara%40cardiff.ac.uk%7Cffa10886d9aa45f2293308da08cdc3f5%7Cbdb74b3095684856bdbf06759778fcbc%7C1%7C0%7C637831977088935528%7CUnknown%7CTWFpbGZsb3d8eyJWIjoiMC4wLjAwMDAiLCJQIjoiV2luMzIiLCJBTiI6Ik1haWwiLCJXVCI6Mn0%3D%7C3000&sdata=IZkhO4Xdj9H75VbAdk%2FHaPm4RjU%2F31V4diScRPXE3rI%3D&reserved=0. Accession number to dataset: E-MTAB-11585. All data are incorporated into the article and its online supplementary material.

## Abbreviations

RA: Rheumatoid arthritis
PNLA: Pinolenic acid
PDK4: Pyruvate dehydrogenase kinase-4
miRNA: MicroRNA
ACR: American College of Rheumatology
EULAR: European Alliance of Associations for Rheumatology
NF-κB: Nuclear factor kappaB
PGE2: Prostaglandin E2
SIGIRR: Single-immunoglobulin interleukin-1 receptor-related molecule
DAP3: Death associated protein-3
WGT: Whole genomic transcriptome
SD: Standard deviation
SEM: Standard error of the mean
EC: Endothelial cell
DC: Dendritic cell
FoxO: Forkhead family of transcription factors
SIRT: Sirtuin
TLRs: Toll like receptor
STAT: Signal transducer and activator of transcription
PUFA: Polyunsaturated fatty acid
MACS: Magnetic activated cell sorting
DMSO: Dimethyl sulphoxide
CVDs: Cardiovascular diseases
KLF15: Kruppel-like factor 15
