# Supplementary material for: Pinolenic acid exhibits anti-inflammatory and anti-atherogenic effects in peripheral blood-derived monocytes from patients with rheumatoid arthritis
Source: Sci Rep. 2022 May 25;12:8807. doi: 10.1038/s41598-022-12763-8 (PMC9133073; doi:10.1038/s41598-022-12763-8)
Supplement: Supplementary file 1 — Supplementary Information 1. [file 41598_2022_12763_MOESM1_ESM.docx]

**Additional file 1**

**Supplementary data S1:** Peripheral blood mononuclear cells (PBMCs) isolation

**Supplementary data S2:** 2.1 Cell surface staining; 2.2. Intracellular staining; 2.3. Flow cytometry analysis

**Supplementary data S3:** RNA extraction methods

**Supplementary data S4:** Upstream regulators as effectors following PNLA treatment

**Supplementary data S5:** CD14+ monocyte percentages were reduced following PNLA treatment

**Supplementary table S1:** Demographic, clinical and laboratory information of RA patients recruited for intracellular cytokines assessment.

**Supplementary table S2:** Demographic, clinical and laboratory information of RA patients recruited for the transcriptome assessment**.**

**Supplementary table S3:** Correlation analysis of the proportion of CD14^+^ expressing cytokines affected by PNLA against variety of clinical indices and lab biomarkers.

**Supplementary table S4:** Top predicted upstream regulators affected by PNLA treatment based on the IPA dataset.

**Supplementary Table S5 (A).** Down-regulated miRNAs

**Supplementary Table S5 (B).** Up-regulated miRNAs

**Supplementary Table S6 (A).** Upregulated protein coding genes

**Supplementary Table S6 (B).** downregulated protein coding genes

**Supplementary figure S1:** Gating strategy for sorting of CD14CD16 monocytes on FACS Sort Aria III.

**Supplementary figure S2:** Heatmap for whole genomic transcriptome of top 500 DEGs.

**Supplementary figure S3:** Principal component analysis of top 50 most DEGs.

**Supplementary figure S4:** Oxidative phosphorylation downstream pathway analysis affected by PNLA

**Supplementary figure S5:** Upstream regulators as effectors of PNLA treatment to

LPS stimulated CD14+ monocytes.

**Supplementary figure S6:** An overview of potential interactions of SIRTs with pathophysiological processes in atherosclerosis
