# Supplementary material for: Pinolenic acid exhibits anti-inflammatory and anti-atherogenic effects in peripheral blood-derived monocytes from patients with rheumatoid arthritis
Source: Sci Rep. 2022 May 25;12:8807. doi: 10.1038/s41598-022-12763-8 (PMC9133073; doi:10.1038/s41598-022-12763-8)
Supplement: Supplementary file 2 — Supplementary Information 2. [file 41598_2022_12763_MOESM2_ESM.docx]

**Additional information**

**Supplementary Data S1**

1.1. Peripheral blood mononuclear cells (PBMCs) isolation

Peripheral blood (PB) was collected in a BD Vacutainer blood collection tube with sodium heparin. PBMCs were prepared from the leukocyte concentrate by Ficoll–Hypaque density gradient centrifugation of fresh blood over Histopaque-1077 (Sigma-Aldrich, UK) from RA patients. The samples then were centrifuged at 400 x g at 22ºC for 30 min (without brakes). The PBMCs layer was carefully harvested using sterile Pasteur pipettes, washed repeatedly with Dulbecco’s PBS (without Ca^2+^ or Mg^2+^) at room temperature (RT) to remove platelet contamination and centrifuged at 250 x g for 10 min at 10°C until the supernatant became clear. To lyse erythrocytes, 1X RBCs lysis buffer (Biolegend, USA) was used after reconstitution in ddH_2_O and incubated with the PBMCs suspension on ice for 15 min as per manufacturer’s instructions. MACS buffer (Miltenyi Biotec) was added to stop the cell lysis as per manufacturer’s instructions and the mixture centrifuged at 350 x g at RT for 5 min. The supernatant was discarded and cell viability was assessed using a trypan blue exclusion assay, where 10 µl of cell suspension was added to 40 µl of a dilute 0.4% (w/v) trypan blue solution (previously prepared in PBS at 1:1 ratio). For cell counting, the haemocytometer method was used. The surface of the haemocytometer was covered in a 5 x 5 grid that can be used to count the cells by adding 10 μl of cell suspension. PBMCs were then aliquoted as 1x10^7^ for the negative selection of monocytes using pan monocytes isolation kit.

**Supplementary Data S2**

2.1 Cell surface staining

After LPS stimulation with/without pre-treatment with PNLA, CD14/CD16 enriched monocytes were collected by detachment of the plastic wells using cold PBS containing 2.5 mM EDTA. The plate was left on ice for 10 min, and clumps of monocytes detached as seen under the microscope. Ca^2^**^+^** ions in EDTA helps in detaching the monocytes and dislodging them in the PBS. The monocytes were then washed using PBS supplemented with 2% HI-FCS, centrifuged at 500 x g for 5 min and non-specific binding to FcR was prevented by incubating 10^6^ cells with 2 ml of FcR human serum immunoglobulin /200 ml PBS buffer for 30 min at 2-8°C. This blocks non-specific binding of the Fc portion of fluorescently conjugated antibodies during staining steps. Then, 2 ml of Zombie Aqua live/dead stain was added to 10^6^ cells/200 ml buffer and incubated in dark for 30 min at RT. Cells were then washed once in FACS staining buffer (0.5% BSA, 5 mM EDTA, 7.5 mM sodium azide in PBS). 2 ml of fluorochrome-conjugated antibodies specific to monocyte surface receptors CD14, and CD16 were added to some of the wells, while identical IgG isotype were added to other wells as a control and incubated for 30-45 min at 2-8°C avoiding direct light (tubes wrapped in foil). 2-4 ml FACS staining buffer was then added, washed twice to remove excess antibody, and centrifuged at 350 x g for 5 min at RT. Supernatants were then discarded, and the pelleted cells resuspended by flicking tubes in 200 µl of 3% paraformaldehyde (PFH) previously prepared in the lab and kept at -4°C. Finally, the cells were vortexed and incubated for 15 min in dark (tubes wrapped in foil) at RT before the next step (section 2.2).

2.2. Intracellular staining

2-4 ml of FACS staining buffer was then added to each tube, mixed well, centrifugated at 350 x g for 5 min at RT, and supernatants discarded. Then, 200 ml of 0.3% saponin (Thermo Fisher Scientific, UK) diluted in PBS was added and incubated for 5 min in dark at RT to allow permeabilization of the monocytes surface. At this step, anti-cytokine panel antibodies or corresponding IgG isotype controls (TNF-α, IL-6, IL-1β, and IL-8) were added and incubated at 2-8°C for 30-45 min. After this incubation, the suspension was washed once with 2-4 ml FACS buffer to remove any excessive staining, supernatants were discarded, and the pellet resuspended in 200 ml of 3% PFH and kept in dark at 2-8°C ready for data acquisition on a flow cytometer (BD LSR-FORTESSA) in the next step (section 2.3).

2.3. Flow cytometry analysis

All acquisition and analysis were performed using the program BD FACSDiva version 8. Compensations and unlabelled (unstained) sample were set before samples acquisition, and the flow rate voltages adjusted on FSC and SSC accordingly. Compensation for fluorochrome settings were generated using compensation beads. Compensation beads were prepared on the day of acquisition by adding 5 µl positive and 5 µl negative bead (BD Biosciences, UK) to each surface or intracellular cytokines antibody in an independent FACS tube and suspended in 200 µl in FACS staining buffer.

**Supplementary Data S3**

3.1. RNA extraction methods

In brief, 24 samples of CD14, CD16 monocytes lysed in buffer RLT (350 µl; provided in the kit) supplemented with 10% of 2-mercaptoethanol (Gibco Life Science, UK) in Eppendorf tubes and kept at -80°C were brought to RT. 350 µl of 70% ethanol (v/v) was then added and mixed well by pipetting up and down. Next, 350 µl of the sample mixture, including any precipitate, was transferred to RNeasy mini spin column placed in 2 ml collection tube (supplied) with the lid closed, centrifuged at 9000 rpm using a microcentrifuge for 15 sec, and the flow through discarded (i.e., nucleic acids are on the column). The rest of the suspension (cell lysate in RLT and ethanol mixture) were added to the column and centrifugation repeated and the flow through discarded, now RNA and DNA on the column. Then, 350 µl of buffer RW1 (wash buffer; provided by the kit) was added to each sample, centrifuged at 9000 rpm in a microcentrifuge for 15 sec, and the flow through discarded. Next, 80 µl of combined 10 µl of DNase I (previously prepared and kept at -20°C) and 70 µl of RDD buffer (kept at 2-8°C) was added to centre of the column and incubated for 15 min at RT. Then, 350 µl of RWI buffer was added, centrifuged at 9000 rpm in a microcentrifuge for 15 sec, and the flow discarded. 500 µl of buffer RPE (wash buffer provided in the kit and constituted with ethanol at 1:5) was added followed by centrifugation in a microcentrifuge at 9000 rpm for 15 sec, and the flow discarded. Finally, 500 µl of RPE was added and centrifuged at 9000 rpm in a microcentrifuge for 2 min, and the flow discarded. After drying the column by centrifugation at 1300 rpm in a microcentrifuge for 1 min, the RNeasy column was then placed into a new 1.5 ml collection tube (supplied in the kit). A 30 µl RNase free water was added directly to the column membrane, the lid closed and centrifuged at 9000 rpm in a microcentrifuge for 1 min to elute RNA. The column was now discarded, and the collection tubes, which contains RNA, were kept at -80°C ready for quality control (QC) check and future library constructions. A quality control check for RNA was performed. QC parameters were applied under the guidance available for library construction using nanodrop spectrophotometer (Thermo Scientific) and Agilent 4200 TapeStation System and RNA ScreenTape kit (Agilent Technologies, UK).

**Nanodrop spectrophotometer**

Following extraction, defrosted total RNA samples were quantified and quality-assessed using the Nanodrop ND-2000 spectrophotometer VX (Thermo Scientific). Nanodrop permits RNA quantification with high accuracy, reproducibility, and low sample consumption (1 μl of total RNA). The values of the absorbance measurements reflect the concentration and purity of the RNA samples. The ratio of absorbance at 260 nm and 230 nm is frequently considered when assessing nucleic acid purity, typical 260/230 ratios are in the range of 2.0-2.2. Also, the 260/280 ratio is used to assess RNA purity with a ratio of 2 generally accepted as pure for RNA.

**Agilent 4200 TapeStation assay**

Total RNA QC check was assessed using Agilent 4200 TapeStation System and RNA ScreenTape kit (Agilent Technologies, UK). The credit card sized, disposable ScreenTape device carries multiple separation lanes for analysing RNA samples, the ScreenTape device has 16 lanes so that each sample is analysed in an individual lane, eliminating contamination. The integrated electrodes apply a current across the screen tape device. The gel through which the samples are analysed is contained within individual columns in the Tape. The TapeStation software automatically determines size, quantity, purity, and RNA integrity number (RIN) thereby reducing errors. Samples were analysed relative to the assay specific ladder.

**Supplementary Data S4**

**Upstream regulators as effectors following PNLA treatment**

IPA also provides a prediction of the likely upstream regulators responsible for the expression changes observed in the experimental dataset (**Supplementary Table S4/Supplementary** **Figures S5).** The activation z-score is used to indicate the likely activation state of the upstream regulator (predicted upregulated molecules have a positive z-score, predicted downregulated molecules have a negative z-score). The p-value of overlap is a measure of the significance of network-regulated gene enrichment within the dataset. DAP3, SIRT3, PPARs, EPA, LIF and STAT3 has been discussed in the main manuscript.

**GSKJ4** is the 3rd most significantly affected regulator. GSK-J4 inhibits LPS-induced TNF-α production in human primary macrophages with an IC50 (the molar concentration required to reduce production by 50%) at 9 μM (Donas *et al*. 2016; Kruidenier *et al.* 2012). GSK-J4 induces endoplasmic reticulum stress-related apoptosis (Yapp *et al.* 2016).

**Let 7** is a family of miRNA was predicted to be activated by PNLA as an upstream regulator. (**Figure 6.21).** shows let-7 networks with molecules and diseases. Certain let-7 family members such as let- 7a and let-7e are associated with early inflammatory and pre-apoptotic pathways, while others such as let-7i and let-7f are involved in later phases of transcription and downstream elements of apoptosis. Such regulatory elements of let 7 miRNAs and the timeline of expression of these miRNAs has subsequent impact on cytokine and chemokine release. Recent bioinformatic predictions have shown several genes, such as FOXP1, AKT2, and PPARGC1A to be targets of different let-7s (let- 7a/7d/7e/7f) (Bernstein *et al.* 2021).

Several factors control the expression of let 7 via regulatory loops. NF-κB reduces let-7 levels. While let-7 can inhibit IL-6 expression that can activate NF-κB and complete a positive feedback loop (Hunter and Jones 2015; Jones 2011). In CIA, disease severity was markedly ameliorated after treatment of let-7g-5p mimics. Interestingly, STAT3 was found to be a target of let-7a and JAK-STAT3 pathway is regulated by miRNA let-7 (Thammaiah and Jayaram 2016). We speculate that let-7 may regulate the activity of monocytes by targeting JAK-STAT3 signalling pathway.

**CHUK**, a serine kinase that is an inhibitor of NF-κB kinase subunit alpha (IKK-α). CHUK is predicted to be inhibited by PNLA. CHUK plays a key role in the negative feedback of NF-κB canonical signalling to limit inflammatory gene activation.

**Supplementary data S5**

**CD14^+^ monocyte percentages were reduced following PNLA treatment**

The levels of activated monocytic cells can be used as an indirect marker of an individual’s inflammatory status. LPS significantly downregulated CD16 expression such that the monocyte subset could not be identified. Hence, analyses focused on CD14**^+^** monocytes.

PNLA reduced the percentage of activated CD14^+^ monocytes relative to the percentage of monocytes that were LPS stimulated **(Figure 1E)**. The number of white blood cells within the BM can be an indication of the inflammatory status of an individual, wild type mice treated with n-3 PUFA were found to have the proportion of white blood cells within their BM reduced in contrast to the mice treated with the vehicle control (Joe 2018). Circulating monocytes numbers are increased in active RA but fall in patients who respond to TNF-blockade. Reduction in monocytes count predicts sustained remission in RA patients treated with anti-TNF therapy (Shipa *et al*. 2021). Kawanaka et al also found that patients with active RA showed higher frequencies of CD14CD16 blood monocytes, with the frequency of these cells decreasing in patients that respond well to therapy (Kawanaka *et al.* 2002). Chara et al in 2015 showed that the increased level of total circulating monocytes; classical and intermediate monocytes in treatment-naive RA patients predicted sub-optimal or non-response to MTX treatment. Further to increase in the number of monocytes in RA, in atherosclerosis the correlation of elevated monocyte counts and higher risk for cardiac events has been confirmed in many reports (Zhuang *et al.* 2017). Additionally, other studies demonstrated that elevated intermediate monocyte counts play a pivotal role in the growth and stability of already existing atherosclerotic plaques or cardiac attacks (Cappellari *et al.* 2017; Ozaki *et al*. 2017).

**References**

Donas C, et al. (2016) The histone demethylase inhibitor GSK-J4 limits inflammation through the induction of a tolerogenic phenotype on DCs. J Autoimmun; **75:105**-117.

Kruidenier L, et al. (2012). A selective jumonji H3K27 demethylase inhibitor modulates the proinflammatory macrophage response. Nature;**488**(7411):404-8.

Yapp, C., Carr, A.J., Price, A., Oppermann, U. and Snelling, S.J., (2016). H3K27me3 demethylases regulate in vitro chondrogenesis and chondrocyte activity in osteoarthritis. Arthritis research & therapy, **18**(1), pp.1-10

Bernstein, D.L., Jiang, X. and Rom, S. (2021). let-7 microRNAs: Their Role in Cerebral and Cardiovascular Diseases, Inflammation, Cancer, and Their Regulation. *Biomedicines* **9**(6), p. 606.

Hunter, C. and Jones, S. (2015). IL-6 as a Keystone Cytokine in Health and Disease. *Nat Immunol***16**:448–57

Jones, S.A., Takeuchi, T., Aletaha, D., Smolen, J., Choy, E.H. and McInnes, I. (2018). Interleukin 6: The biology behind the therapy. *Considerations in Medicine* **2**(1):2.

Thammaiah, C.K. and Jayaram, S. (2016). Role of let-7 family microRNA in breast cancer. *Non-coding RNA Research* 1(1), pp. 77–82.

Brennan, E. et al. (2017). Protective Effect of let-7 miRNA Family in Regulating Inflammation in Diabetes- Associated Atherosclerosis. *Diabetes* **66**(8), p. 2266.

Shipa, M.R.A., Amarnani, R., Yeoh, S.-A., Mainuddin, M.D. and Ehrenstein, M.R. (2021). Early reduction in circulating monocyte count predicts maintenance of remission in patients with rheumatoid arthritis treated with anti-TNF therapy. Annals of the Rheumatic Diseases, p. annrheumdis-220642.

Kawanaka, N., Yamamura, M., Aita, T., Morita, Y., Okamoto, A., Kawashima, M., Iwahashi, M., Ueno, A., Ohmoto, Y. and Makino, H., (2002). CD14+, CD16+ blood monocytes and joint inflammation in rheumatoid arthritis. Arthritis & Rheumatism, 46(10), pp.2578-2586.

Zhuang, J., Han, Y., Xu, D., Zhu, G., Singh, S., Chen, L., Zhu, M., Chen, W., Xu, Y. and Li, X., (2017). Comparison of circulating dendritic cell and monocyte subsets at different stages of atherosclerosis: insights from optical coherence tomography. BMC cardiovascular disorders, 17(1), pp.1-10.

Cappellari, R., D'Anna, M., Bonora, B.M., Rigato, M., Cignarella, A., Avogaro, A. and Fadini, G.P., (2017). Shift of monocyte subsets along their continuum predicts cardiovascular outcomes. Atherosclerosis, 266, pp.95-102.

Ozaki, Y., Imanishi, T., Hosokawa, S., Nishiguchi, T., Taruya, A., Tanimoto, T., Kuroi, A., Yamano, T., Matsuo, Y., Ino, Y. and Kitabata, H., (2017). Association of toll-like receptor 4 on human monocyte subsets and vulnerability characteristics of coronary plaque as assessed by 64-slice multidetector computed tomography. Circulation Journal, pp.CJ-16.
