# Supplementary material for: Pinolenic acid exhibits anti-inflammatory and anti-atherogenic effects in peripheral blood-derived monocytes from patients with rheumatoid arthritis
Source: Sci Rep. 2022 May 25;12:8807. doi: 10.1038/s41598-022-12763-8 (PMC9133073; doi:10.1038/s41598-022-12763-8)
Supplement: Supplementary file 3 — Supplementary Figure 1. [file 41598_2022_12763_MOESM3_ESM.pptx]

## Slide 1
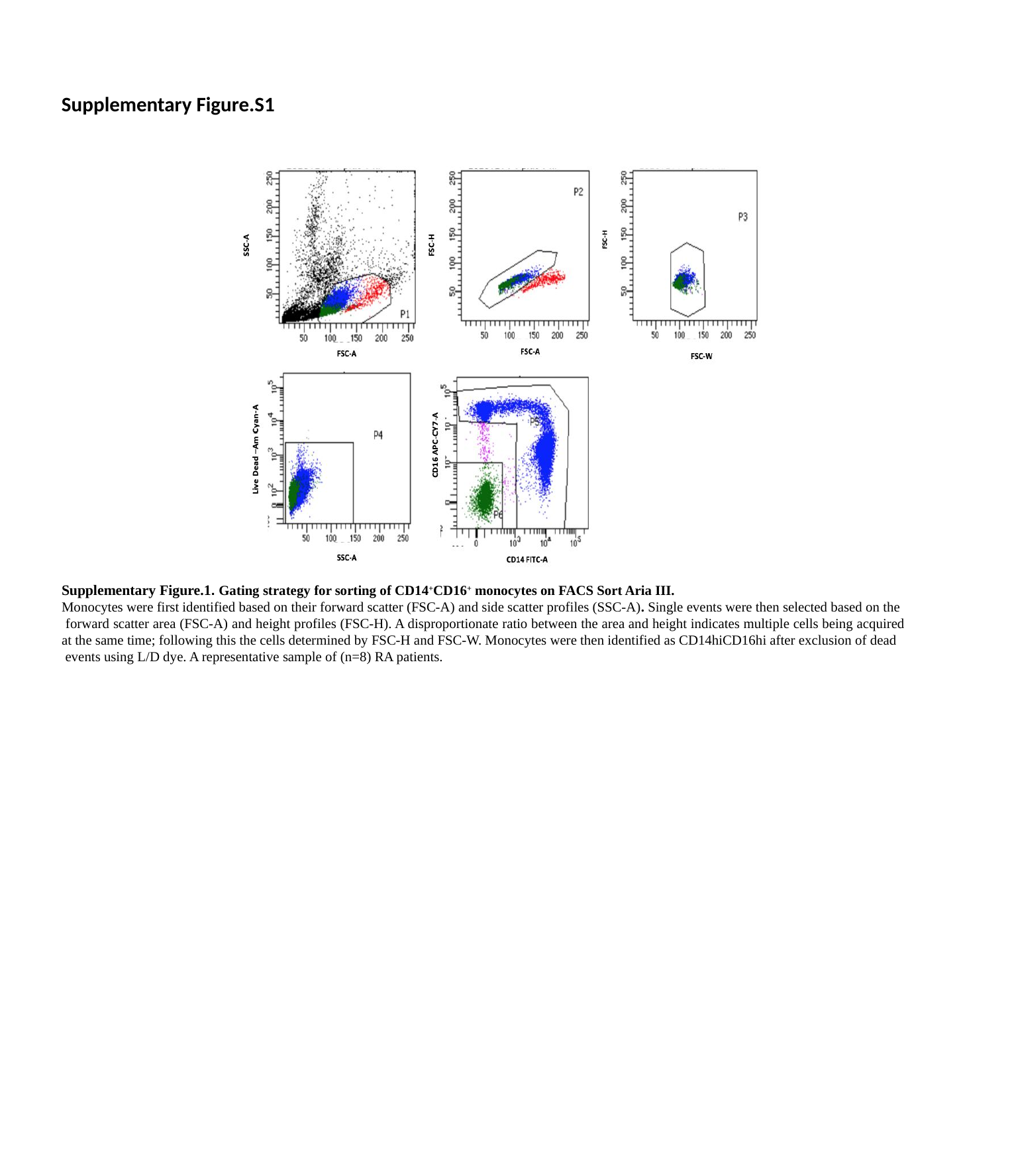

Supplementary Figure.S1
Supplementary Figure.1. Gating strategy for sorting of CD14+CD16+ monocytes on FACS Sort Aria III.
Monocytes were first identified based on their forward scatter (FSC-A) and side scatter profiles (SSC-A). Single events were then selected based on the
 forward scatter area (FSC-A) and height profiles (FSC-H). A disproportionate ratio between the area and height indicates multiple cells being acquired at the same time; following this the cells determined by FSC-H and FSC-W. Monocytes were then identified as CD14hiCD16hi after exclusion of dead
 events using L/D dye. A representative sample of (n=8) RA patients.
