# Supplementary material for: Pinolenic acid exhibits anti-inflammatory and anti-atherogenic effects in peripheral blood-derived monocytes from patients with rheumatoid arthritis
Source: Sci Rep. 2022 May 25;12:8807. doi: 10.1038/s41598-022-12763-8 (PMC9133073; doi:10.1038/s41598-022-12763-8)
Supplement: Supplementary file 4 — Supplementary Figure 2. [file 41598_2022_12763_MOESM4_ESM.pptx]

## Slide 1
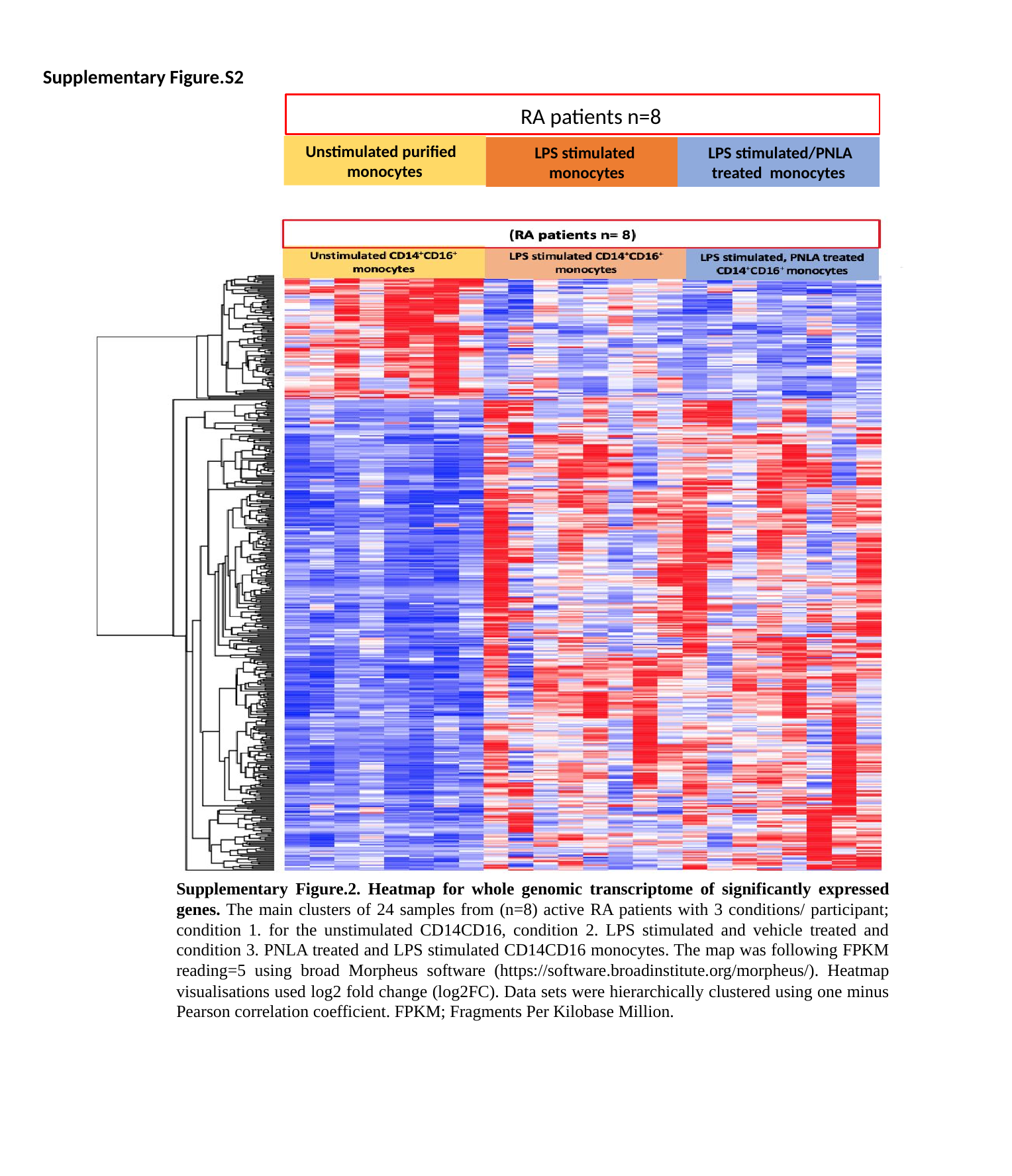

Supplementary Figure.S2
 RA patients n=8
 Unstimulated purified monocytes
LPS stimulated
monocytes
 LPS stimulated/PNLA treated monocytes
Supplementary Figure.2. Heatmap for whole genomic transcriptome of significantly expressed genes. The main clusters of 24 samples from (n=8) active RA patients with 3 conditions/ participant; condition 1. for the unstimulated CD14CD16, condition 2. LPS stimulated and vehicle treated and condition 3. PNLA treated and LPS stimulated CD14CD16 monocytes. The map was following FPKM reading=5 using broad Morpheus software (https://software.broadinstitute.org/morpheus/). Heatmap visualisations used log2 fold change (log2FC). Data sets were hierarchically clustered using one minus Pearson correlation coefficient. FPKM; Fragments Per Kilobase Million.
