# Supplementary material for: Pinolenic acid exhibits anti-inflammatory and anti-atherogenic effects in peripheral blood-derived monocytes from patients with rheumatoid arthritis
Source: Sci Rep. 2022 May 25;12:8807. doi: 10.1038/s41598-022-12763-8 (PMC9133073; doi:10.1038/s41598-022-12763-8)
Supplement: Supplementary file 5 — Supplementary Figure 3. [file 41598_2022_12763_MOESM5_ESM.pptx]

## Slide 1
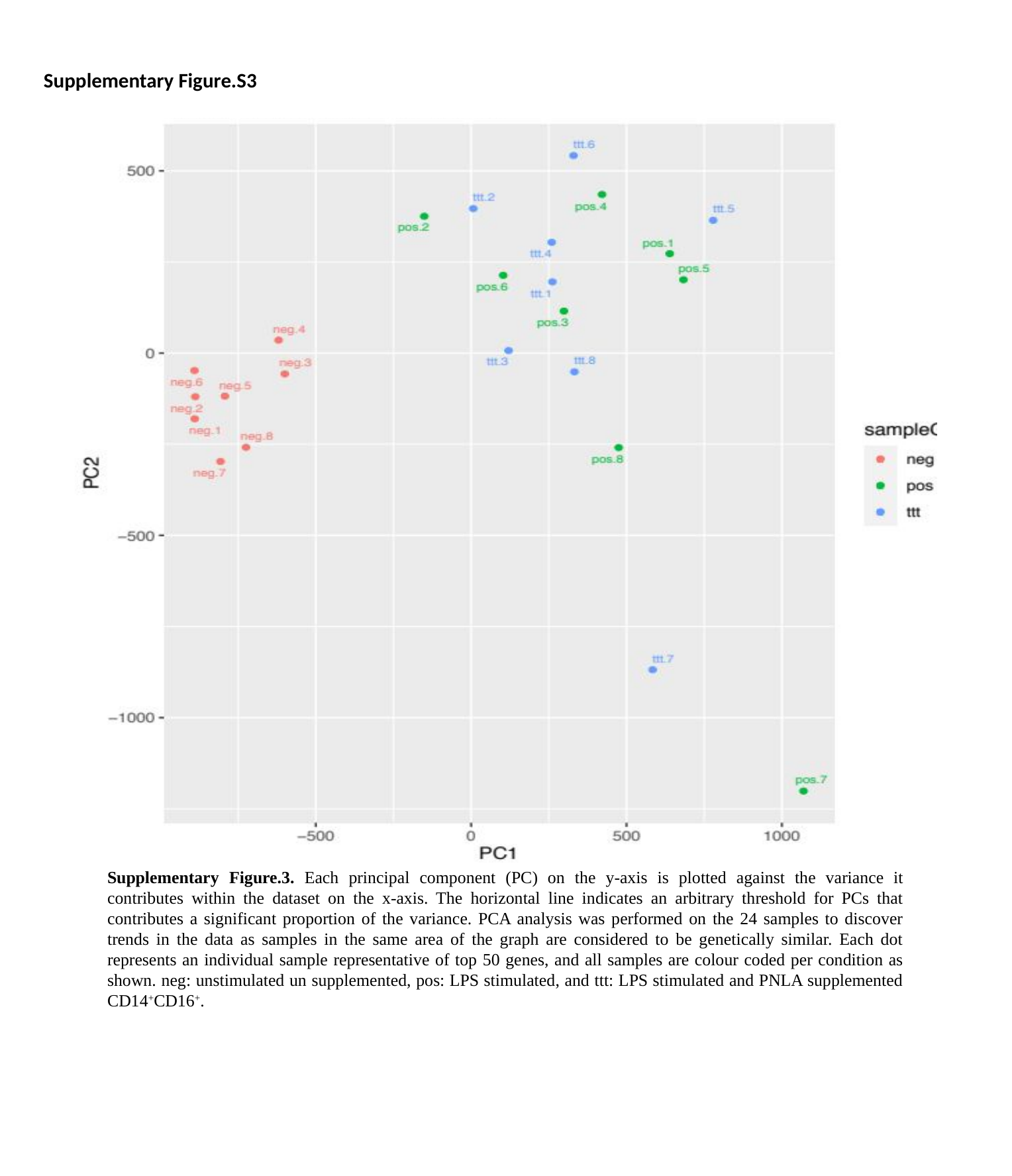

Supplementary Figure.S3
Supplementary Figure.3. Each principal component (PC) on the y-axis is plotted against the variance it contributes within the dataset on the x-axis. The horizontal line indicates an arbitrary threshold for PCs that contributes a significant proportion of the variance. PCA analysis was performed on the 24 samples to discover trends in the data as samples in the same area of the graph are considered to be genetically similar. Each dot represents an individual sample representative of top 50 genes, and all samples are colour coded per condition as shown. neg: unstimulated un supplemented, pos: LPS stimulated, and ttt: LPS stimulated and PNLA supplemented CD14+CD16+.
