# Supplementary material for: Pinolenic acid exhibits anti-inflammatory and anti-atherogenic effects in peripheral blood-derived monocytes from patients with rheumatoid arthritis
Source: Sci Rep. 2022 May 25;12:8807. doi: 10.1038/s41598-022-12763-8 (PMC9133073; doi:10.1038/s41598-022-12763-8)
Supplement: Supplementary file 6 — Supplementary Figure 4. [file 41598_2022_12763_MOESM6_ESM.pptx]

## Slide 1
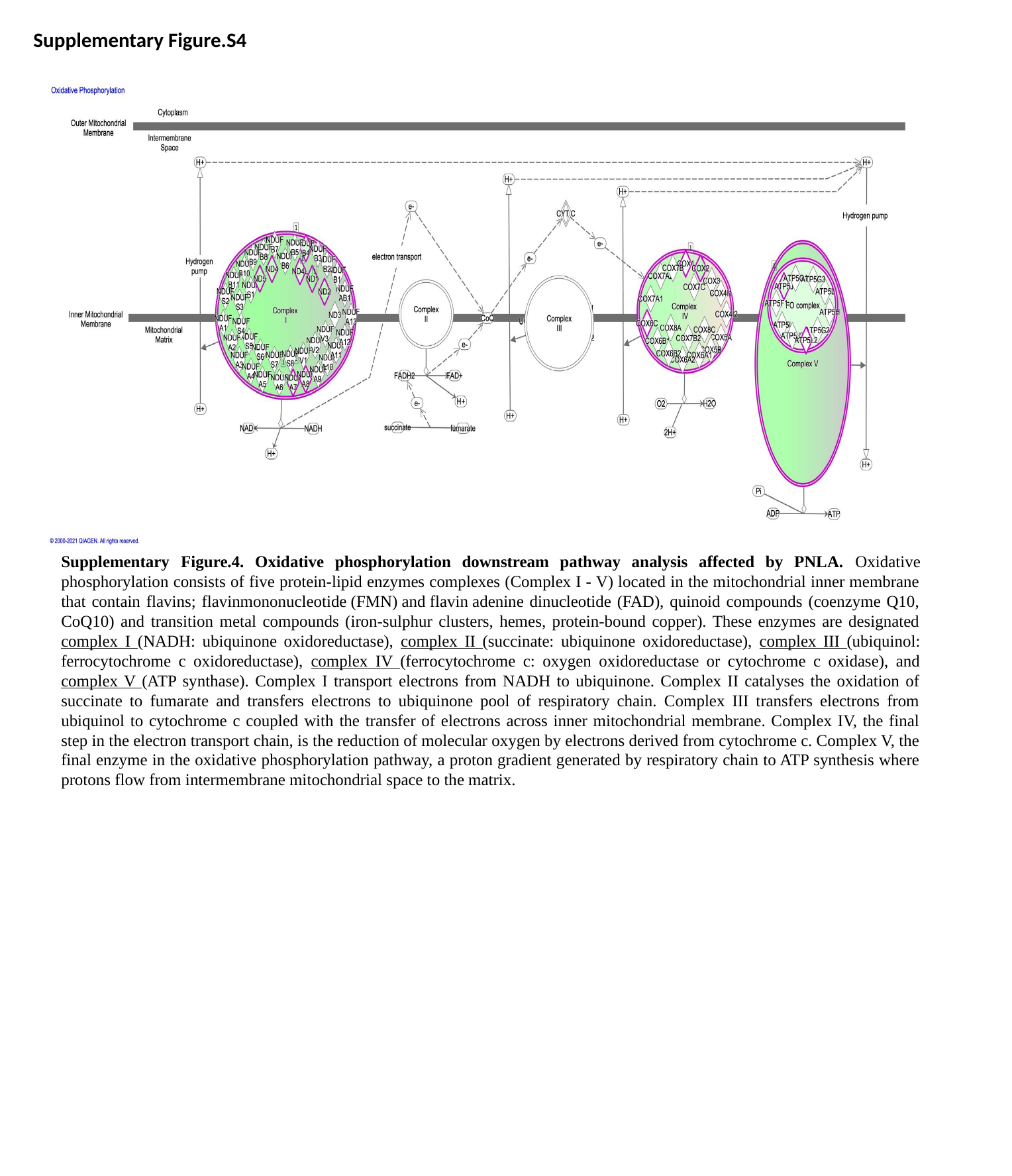

Supplementary Figure.S4
Supplementary Figure.4. Oxidative phosphorylation downstream pathway analysis affected by PNLA. Oxidative phosphorylation consists of five protein-lipid enzymes complexes (Complex I - V) located in the mitochondrial inner membrane that contain flavins; flavinmononucleotide (FMN) and flavin adenine dinucleotide (FAD), quinoid compounds (coenzyme Q10, CoQ10) and transition metal compounds (iron-sulphur clusters, hemes, protein-bound copper). These enzymes are designated complex I (NADH: ubiquinone oxidoreductase), complex II (succinate: ubiquinone oxidoreductase), complex III (ubiquinol: ferrocytochrome c oxidoreductase), complex IV (ferrocytochrome c: oxygen oxidoreductase or cytochrome c oxidase), and complex V (ATP synthase). Complex I transport electrons from NADH to ubiquinone. Complex II catalyses the oxidation of succinate to fumarate and transfers electrons to ubiquinone pool of respiratory chain. Complex III transfers electrons from ubiquinol to cytochrome c coupled with the transfer of electrons across inner mitochondrial membrane. Complex IV, the final step in the electron transport chain, is the reduction of molecular oxygen by electrons derived from cytochrome c. Complex V, the final enzyme in the oxidative phosphorylation pathway, a proton gradient generated by respiratory chain to ATP synthesis where protons flow from intermembrane mitochondrial space to the matrix.
