# Supplementary material for: Pinolenic acid exhibits anti-inflammatory and anti-atherogenic effects in peripheral blood-derived monocytes from patients with rheumatoid arthritis
Source: Sci Rep. 2022 May 25;12:8807. doi: 10.1038/s41598-022-12763-8 (PMC9133073; doi:10.1038/s41598-022-12763-8)
Supplement: Supplementary file 7 — Supplementary Figure 5. [file 41598_2022_12763_MOESM7_ESM.pptx]

## Slide 1
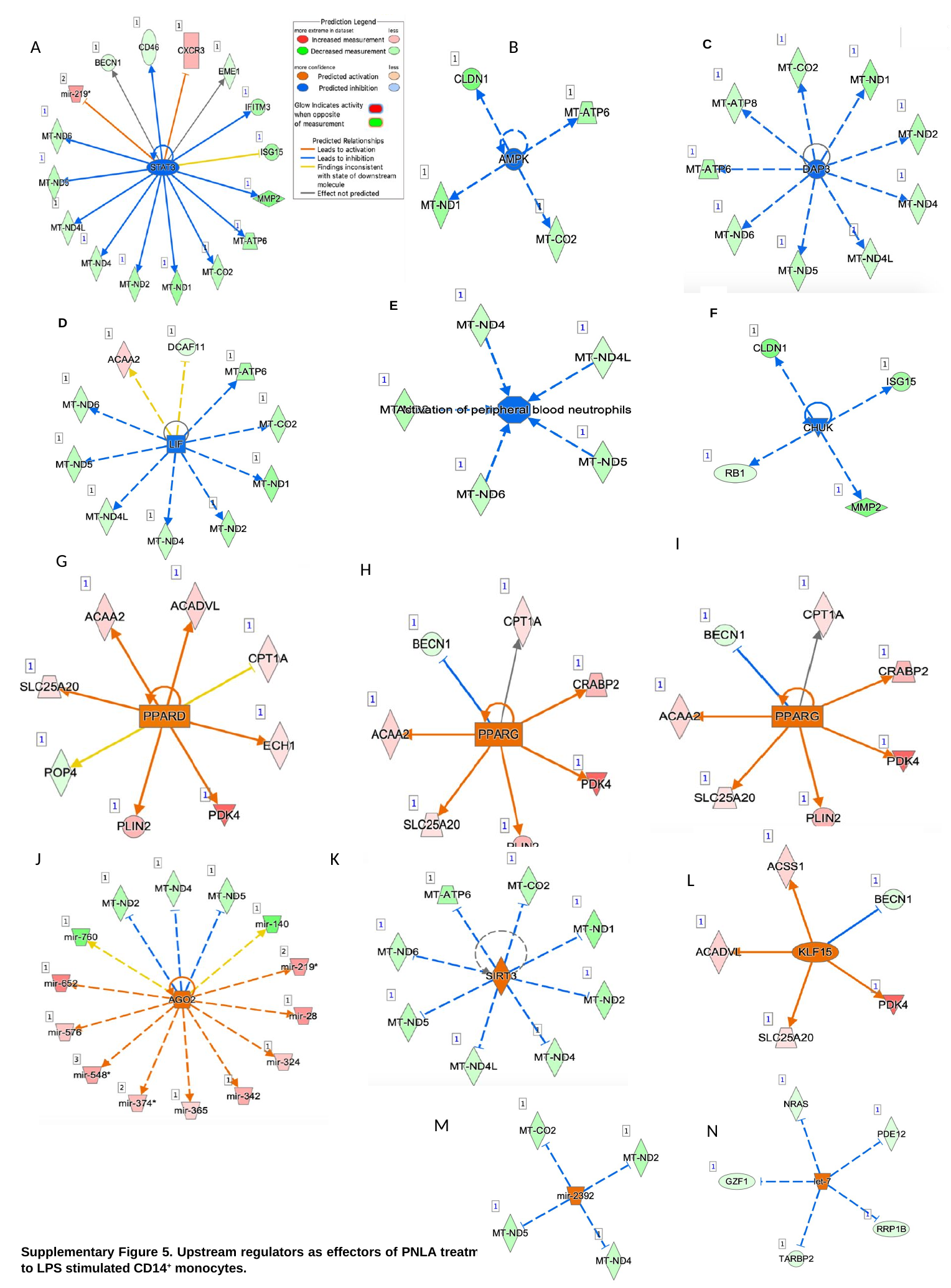

C
A
B
E
F
D
I
G
H
K
J
L
M
N
Supplementary Figure 5. Upstream regulators as effectors of PNLA treatment to LPS stimulated CD14+ monocytes.
