# Supplementary material for: Pinolenic acid exhibits anti-inflammatory and anti-atherogenic effects in peripheral blood-derived monocytes from patients with rheumatoid arthritis
Source: Sci Rep. 2022 May 25;12:8807. doi: 10.1038/s41598-022-12763-8 (PMC9133073; doi:10.1038/s41598-022-12763-8)
Supplement: Supplementary file 8 — Supplementary Figure 6. [file 41598_2022_12763_MOESM8_ESM.pptx]

## Slide 1
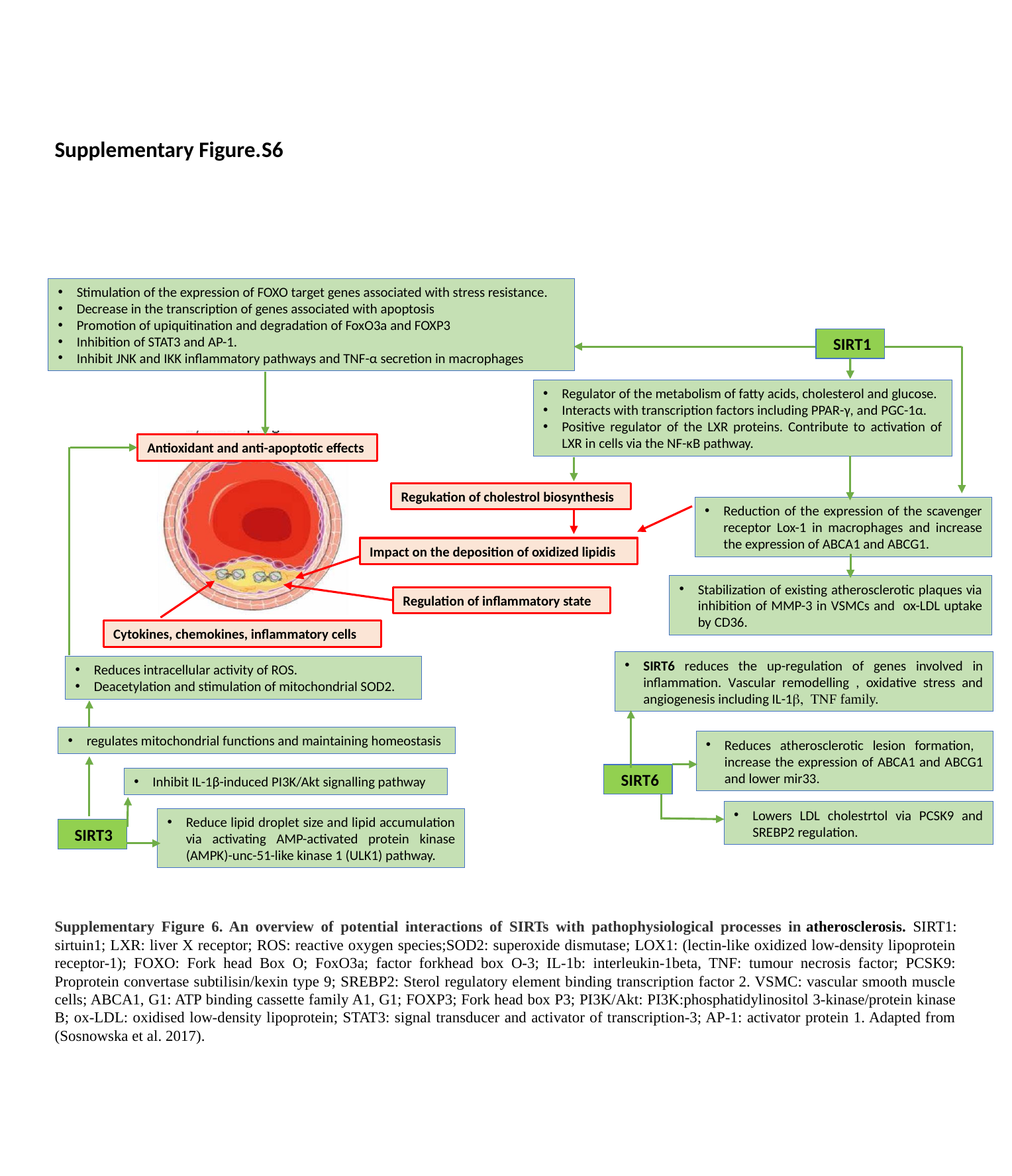

Supplementary Figure.S6
Stimulation of the expression of FOXO target genes associated with stress resistance.
Decrease in the transcription of genes associated with apoptosis
Promotion of upiquitination and degradation of FoxO3a and FOXP3
Inhibition of STAT3 and AP-1.
Inhibit JNK and IKK inflammatory pathways and TNF-α secretion in macrophages
 SIRT1
Regulator of the metabolism of fatty acids, cholesterol and glucose.
Interacts with transcription factors including PPAR-γ, and PGC-1α.
Positive regulator of the LXR proteins. Contribute to activation of LXR in cells via the NF-κB pathway.
Antioxidant and anti-apoptotic effects
Regukation of cholestrol biosynthesis
Reduction of the expression of the scavenger receptor Lox-1 in macrophages and increase the expression of ABCA1 and ABCG1.
Impact on the deposition of oxidized lipidis
Stabilization of existing atherosclerotic plaques via inhibition of MMP-3 in VSMCs and ox-LDL uptake by CD36.
Regulation of inflammatory state
Cytokines, chemokines, inflammatory cells
SIRT6 reduces the up-regulation of genes involved in inflammation. Vascular remodelling , oxidative stress and angiogenesis including IL-1b, TNF family.
Reduces intracellular activity of ROS.
Deacetylation and stimulation of mitochondrial SOD2.
regulates mitochondrial functions and maintaining homeostasis
Reduces atherosclerotic lesion formation, increase the expression of ABCA1 and ABCG1 and lower mir33.
 SIRT6
Inhibit IL-1β-induced PI3K/Akt signalling pathway
Lowers LDL cholestrtol via PCSK9 and SREBP2 regulation.
Reduce lipid droplet size and lipid accumulation via activating AMP-activated protein kinase (AMPK)-unc-51-like kinase 1 (ULK1) pathway.
 SIRT3
Supplementary Figure 6. An overview of potential interactions of SIRTs with pathophysiological processes in atherosclerosis. SIRT1: sirtuin1; LXR: liver X receptor; ROS: reactive oxygen species;SOD2: superoxide dismutase; LOX1: (lectin-like oxidized low-density lipoprotein receptor-1); FOXO: Fork head Box O; FoxO3a; factor forkhead box O-3; IL-1b: interleukin-1beta, TNF: tumour necrosis factor; PCSK9: Proprotein convertase subtilisin/kexin type 9; SREBP2: Sterol regulatory element binding transcription factor 2. VSMC: vascular smooth muscle cells; ABCA1, G1: ATP binding cassette family A1, G1; FOXP3; Fork head box P3; PI3K/Akt: PI3K:phosphatidylinositol 3-kinase/protein kinase B; ox-LDL: oxidised low-density lipoprotein; STAT3: signal transducer and activator of transcription-3; AP-1: activator protein 1. Adapted from (Sosnowska et al. 2017).
