# Supplementary material for: Pinolenic acid exhibits anti-inflammatory and anti-atherogenic effects in peripheral blood-derived monocytes from patients with rheumatoid arthritis
Source: Sci Rep. 2022 May 25;12:8807. doi: 10.1038/s41598-022-12763-8 (PMC9133073; doi:10.1038/s41598-022-12763-8)
Supplement: Supplementary file 9 — Supplementary Tables. [file 41598_2022_12763_MOESM9_ESM.docx]

**Additional information**

**Supplementary Table S1.** **Demographic, clinical and laboratory information of RA patients recruited for intracellular cytokines assessment.**

| Subject | Age (y) | Gender | DD (y) | RF | ACPA | ESR | CRP | DAS28 | Treatment |
| --- | --- | --- | --- | --- | --- | --- | --- | --- | --- |
| RA1 | 63 | F | 6 | +ve | +ve | 10 | 2 | 3.4 | Rituximab + MTX |
| RA2 | 52 | F | 18 | +ve | +ve | 15 | 7 | 3.6 | Infliximab |
| RA3 | 68 | F | 22 | +ve | -ve | 2 | 2 | 5.2 | Rituximab |
| RA4 | 78 | F | 10 | +ve | -ve | 4 | 3 | 2.8 | Tocilizumab |
| RA5 | 63 | F | 3 | -ve | -ve | 16 | 11 | 5.2 | Tocilizumab |
| RA6 | 70 | F | 22 | +ve | -ve | 60 | 25 | 5.2 | Infliximab |
| RA7 | 45 | F | 3 | -ve | +ve | 5 | 4 | 3.3 | Rituximab + MTX |
| RA8 | 35 | M | 4 | -ve | -ve | 12 | 11 | 5 | Rituximab + MTX |
| RA9 | 78 | F | 13 | +ve | +ve | 10 | 1 | 3.4 | Infliximab + MTX |
| RA10 | 58 | F | 4 | +ve | +ve | 9 | 1 | 5 | Rituximab + MTX |
| RA11 | 43 | M | 21 | -ve | -ve | 2 | 10 | 3 | Infliximab |
| RA12 | 75 | F | 18 | -ve | -ve | 3 | 1 | 4.3 | Infliximab + MTX |
| RA13 | 74 | F | 13 | +ve | NA | 10 | 25 | 1.49 | Rituximab + MTX |
| RA14 | 70 | F | 12 | +ve | +ve | 7 | 1 | 2.8 | Tocilizumab |
| RA15 | 45 | M | 14 | -ve | -ve | 51 | 86 | 5.2 | Rituximab+ MTX |
| RA16 | 78 | F | 11 | -ve | -ve | 5 | 9 | 4 | Rituximab |
| RA17 | 63 | F | 6 | +ve | +ve | 2 | 1 | 3.6 | Rituximab |
| RA18 | 57 | F | 13 | -ve | NA | 12 | 3 | 3.5 | Rituximab, MMF |
| RA19 | 65 | F | 21 | -ve | NA | 4 | 0 | 2 | Tocilizumab |
| RA20 | 60 | M | 10 | +ve | +ve | 2 | 0 | 2 | Rituximab |
| SD | 12.71 |  | 12.71 |  |  | 15.56 | 19.33 | 1.16 |  |

F; female, M; male, DD; disease duration, y; years, ACPA; Anti-citrullinated protein antibody, RF; rheumatoid factor, ESR; erythrocytes sedimentation rates the normal ESR is < 20mm/hour for female and <10 mm/hour for male CRP; C-reactive protein and the normal CRP is < 4.9 mg/l. MTX; methotrexate, MMF; mycophenolate mofetil, y; years, NA: not available. SD: standard deviation, Patients in remission is shown in red

**Supplementary Table S2.** **Demographic, clinical and laboratory information of RA patients recruited for the transcriptome assessment.**

| Subject | Gender | Age (y) | DD (y) | RF | ACPA | DAS | CRP | ESR | Treatment |
| --- | --- | --- | --- | --- | --- | --- | --- | --- | --- |
| RA1 | F | 57 | 18 | - ve | + ve | 5.1 | 32 | 36 | Abatacept |
| RA2 | F | 55 | 22 | + ve | + ve | 5 | 1 | 5 | Infliximab |
| RA3 | F | 56 | 12 | +ve | + ve | 5.2 | 17 | 10 | Rituxamab |
| RA4 | F | 58 | 14 | +ve | + ve | 5 | 0 | 8 | Rituxamab |
| RA5 | M | 68 | 11 | +ve | + ve | 5 | 6 | 2 | Rituxamab, MTX |
| RA6 | F | 63 | 13 | +ve | + ve | 4.8 | 2 | 8 | Rituxamab, MTX |
| RA7 | F | 70 | 17 | +ve | + ve | 5.7 | 11 | 21 | Rituxamab |
| RA8 | M | 64 | 12 | +ve | + ve | 5.5 | 34 | 46 | Rituxamab, MTX |
| SD |  | 5.70 | 3.79 |  |  | 0.29 | 13.65 | 16.026 |  |

**Supplementary Table S3.** **Correlation analysis of the percentage reduction of CD14+ expressing cytokines against variety of clinical indices and lab biomarkers. Data passed the normality. R-value on top with corresponding p-values on the subsequent rows below.**

|  | **% reduction in IL-6 expression** | **% reduction in TNF-α expression** | **% reduction in IL-1β expression** | **Age** | **DD** | **ESR** | **CRP** | **DAS28** |
| --- | --- | --- | --- | --- | --- | --- | --- | --- |
| **Age** | -.058 | -.169 | .270 |  |  |  |  |  |
|  | .808 | .475 | .250 |  |  |  |  |  |
| **DD** | .122 | -.022 | -.138 | .205 |  |  |  |  |
|  | .610 | .927 | .563 | .387 |  |  |  |  |
| **ESR** | .090 | -.117 | .477* | -.164 | -.064 |  |  |  |
|  | .706 | .622 | .034* | .490 | .789 |  |  |  |
| **CRP** | .003 | -.277 | .308 | -.245 | .054 | .613** |  |  |
|  | .990 | .238 | .187 | .298 | .821 | .004** |  |  |
| **DAS28** | .038 | -.063 | .214 | -.185 | .011 | .435 | .400 |  |
|  | .872 | .791 | .366 | .434 | .962 | .056 | .081 |  |

**DD; disease duration, ESR; erythrocytes sedimentation rate, CRP; C-reactive protein, and DAS28; disease activity score 28. Red shown * highlight the significant correlations** No statistically significant correlation was found between these variables and percentage reduction in cytokine expression apart for IL-1β and ESR. For the latter, the difference was not statistically significant after Bonferroni correction for multiple comparisons

**Supplementary Table S4.** **Top predicted upstream regulators affected by PNLA treatment** **based on the IPA dataset**

| **Upstream regulator** | **Molecule Type** | **Activation z-score** | **p-value of overlap** |
| --- | --- | --- | --- |
| **DAP3** | other | -3 | 2.31E-14 |
| **Actinonine** | chemical reagent | 3 | 5.04E-11 |
| **GSKJ4** | chemical reagent | 3.317 | 3.01E-08 |
| **AGO2** | translation regulator | 2.646 | 2.16E-08 |
| **SIRT3** | enzyme | 2.818 | 3.03E-06 |
| **EPA** | chemical drug | 2.425 | 4.16E-03 |
| **LIF** | cytokine | -1.897 | 1.74E-03 |
| **PPAR-γ** | transcription regulator | 2.355 | 1.00E-03 |
| **KLF15** | transcription regulator | 2.226 | 1.07E03 |
| **PPAR-δ** | transcription regulator | 1.651 | 4.78E-02 |
| **STAT3** | transcription regulator | -3.203 | 3.37E-02 |
| **AMPK** | complex | -2 | 1.09E-02 |
| **VEGF** | Group | -1.969 | 1.00E-02 |
| **MYC** | transcription regulator | -1.822 | 1.00E-02 |
| **CHUK** | kinase | -1.964 | 3.96E-01 |

**Supplementary Table S5(A). Down-regulated miRNAs**

| **miRNA symbol** | **P value** | **log2FoldChange** |
| --- | --- | --- |
| **MIR637** | 0.00634466 | -1.7466434 |
| **MIR4326** | 0.01094654 | -1.0103594 |
| **MIR6886** | 0.02544526 | -2.4344905 |
| **MIR1909** | 0.03026114 | -0.5482978 |
| **MIR671** | 0.03855069 | -0.3497858 |
| **MIR7111** | 0.04708788 | -0.7253948 |

| **miRNA symbol**  **Supplementary Table S5(B). Up-regulated miRNAs** | **P value** | **log2FoldChange** |
| --- | --- | --- |
| **MIR8066** | 0.00011357 | 3.64199116 |
| **MIR1276** | 0.00068454 | 4.55094396 |
| **MIR3173** | 0.00110552 | 1.97460738 |
| **MIR664B** | 0.00153033 | 2.99076371 |
| **MIR6773** | 0.00233385 | 3.63985987 |
| **MIR6778** | 0.00311499 | 4.28591091 |
| **MIR374C** | 0.00311767 | 1.39533476 |
| **MIR374B** | 0.00345498 | 1.38208333 |
| **MIR3161** | 0.00427845 | 1.24687388 |
| **MIR219B** | 0.00459433 | 2.06906127 |
| **MIR3922** | 0.00475857 | 2.27944349 |
| **MIR219A2** | 0.00489499 | 1.91298973 |
| **MIR1914** | 0.00518867 | 1.27590652 |
| **MIR505** | 0.00605733 | 2.10537396 |
| **MIR3140** | 0.00680206 | 1.7364798 |
| **MIR941-3** | 0.00757542 | 1.69321925 |
| **MIR324** | 0.00761552 | 1.15616334 |
| **MIR4722** | 0.00929873 | 1.51670221 |
| **MIR4755** | 0.00933625 | 1.27252376 |
| **MIR3176** | 0.0098648 | 1.436178 |
| **MIR3978** | 0.01044756 | 1.75906105 |
| **MIR4435-2** | 0.01158254 | 3.95408034 |
| **MIR4440** | 0.01358341 | 2.9973427 |
| **MIR1470** | 0.01443236 | 3.92022505 |
| **MIR570** | 0.01560925 | 0.86245152 |
| **MIR6719** | 0.0184946 | 2.4778449 |
| **MIR365B** | 0.01973051 | 0.8725209 |
| **MIR6516** | 0.01995768 | 0.95898745 |
| **MIR28** | 0.01996707 | 2.18139375 |
| **MIR1260B** | 0.02060822 | 0.90146242 |
| **MIR6744** | 0.02068237 | 2.1691624 |
| **MIR4434** | 0.02440112 | 1.15863809 |
| **MIR342** | 0.02533851 | 1.74143132 |
| **MIR620** | 0.02730656 | 1.53792619 |
| **MIR6755** | 0.02833511 | 1.46159167 |
| **MIR3679** | 0.03570583 | 1.29447685 |
| **MIR3188** | 0.03579885 | 1.95841277 |
| **MIR7107** | 0.03760526 | 0.76246923 |
| **MIR32** | 0.04072735 | 2.48790672 |
| **MIR4725** | 0.04135483 | 0.96185387 |
| **MIR5088** | 0.04231274 | 1.47488951 |
| **MIR378H** | 0.04286645 | 1.24063468 |
| **MIR7150** | 0.04294533 | 1.80549093 |
| **MIR6763** | 0.04328839 | 1.63076905 |
| **MIR626** | 0.04458306 | 1.48601166 |

**Supplementary Table S6(A). Upregulated protein coding genes**

| Gene symbol | P value | log2FoldChange |
| --- | --- | --- |
| LY6G5B | 0.00034881 | 0.70755274 |
| PDK4 | 0.00036415 | 3.18958087 |
| BRF1 | 0.00325595 | 0.4337583 |
| ACAA2 | 0.00343723 | 0.91003443 |
| ZBTB34 | 0.00427356 | 0.40754133 |
| ACADVL | 0.00443301 | 0.93393128 |
| AC007375.2 | 0.00654865 | 0.84877037 |
| SPINK4 | 0.00699286 | 2.33516822 |
| CPT1A | 0.00844875 | 0.69292304 |
| AC090227.2 | 0.0102456 | 0.7761555 |
| GRIK1 | 0.01072531 | 1.04080934 |
| HSD17B8 | 0.01459802 | 0.63690587 |
| AC008481.3 | 0.01575296 | 0.67745158 |
| AP4B1 | 0.01597519 | 0.35373153 |
| PLIN2 | 0.01695979 | 1.52650127 |
| NPEPL1 | 0.0178674 | 0.34366431 |
| KDM4C | 0.01845432 | 0.4053604 |
| CCDC88B | 0.01847757 | 0.92737706 |
| IRF3 | 0.01931028 | 0.38814126 |
| HSPB9 | 0.02058611 | 1.00732108 |
| AC012651.1 | 0.02101049 | 0.40204391 |
| AL133500.1 | 0.02345169 | 0.53492741 |
| MRE11 | 0.02539087 | 0.33449725 |
| XPA | 0.02547796 | 0.40955563 |
| RNF166 | 0.02632569 | 0.60565988 |
| MTG1 | 0.02779212 | 0.41040856 |
| SOX15 | 0.02786463 | 0.51263859 |
| ZNF48 | 0.02819684 | 1.1309529 |
| ANKRD23 | 0.03246504 | 0.6371512 |
| C12orf75 | 0.03294798 | 1.55318072 |
| CCER2 | 0.03297806 | 1.2292063 |
| CCDC194 | 0.0345181 | 1.23143686 |
| SLC25A42 | 0.03850798 | 1.12134178 |
| GHRL | 0.03879516 | 0.4293575 |
| ALG13 | 0.03960082 | 0.41540235 |
| CRABP2 | 0.04073619 | 1.4878064 |
| MTRNR2L8 | 0.04206278 | 0.50185453 |
| ROM1 | 0.04371743 | 0.47498045 |
| ST14 | 0.04414583 | 0.94104069 |
| SIGIRR | 0.04453364 | 1.12389203 |
| ZBTB40 | 0.04498858 | 0.17567415 |
| AKR1B1 | 0.04687118 | 0.62649418 |
| RCN3 | 0.04840635 | 0.57764257 |
| CLDND2 | 0.048774 | 1.63566507 |
| KLHDC4 | 0.04980061 | 0.6491871 |
| ETFA | 0.04981843 | 0.24752697 |
| JMJD7-PLA2G4B | 0.05002327 | 0.48261373 |
| \|  \| **SLC25A20** \|  \| \| --- \| --- \| --- \| | 0.05004554 | 0.50986113 |

**Supplementary Table S6(B) downregulated protein coding genes**

| Gene symbol | P value | log2FoldChange |
| --- | --- | --- |
| SPCS1 | 0.00457316 | -0.3889066 |
| RHNO1 | 0.00772516 | -0.445853 |
| MRPL9 | 0.00897261 | -0.3562887 |
| MT-ND1 | 0.0111281 | -1.1519433 |
| HAUS2 | 0.01408627 | -0.2589403 |
| AC113189.9 | 0.02021096 | -0.6329407 |
| HSPA1L | 0.02261342 | -0.8068353 |
| MT-CO2 | 0.02426093 | -0.8215064 |
| CHCHD4 | 0.02494514 | -0.5253673 |
| NIT1 | 0.02578718 | -0.2936841 |
| PAIP1 | 0.02652437 | -0.2093219 |
| OTUB1 | 0.02863435 | -0.2062052 |
| MEN1 | 0.02892991 | -0.2316949 |
| OTUB1 | 0.02863435 | -0.2062052 |
| MEN1 | 0.02892991 | -0.2316949 |
| ATMIN | 0.03039483 | -0.2434687 |
| MT-ATP6 | 0.03071407 | -0.9914844 |
| ENOX2 | 0.0310368 | -0.2614889 |
| LSM1 | 0.03115697 | -0.2821932 |
| ARL2BP | 0.03163195 | -0.2534689 |
| MT-ND5 | 0.0331925 | -0.8105088 |
| FZD2 | 0.03337634 | -1.0574003 |
| EEFSEC | 0.03359343 | -0.2823531 |
| SHARPIN | 0.03434721 | -0.3720591 |
| MEA1 | 0.03439924 | -0.2134155 |
| AC007731.4 | 0.03477517 | -0.4368948 |
| PPP1R7 | 0.03567901 | -0.2324188 |
| JMJD4 | 0.03582529 | -0.3678366 |
| ZNF428 | 0.03871094 | -0.703142 |
| SLC10A3 | 0.03940111 | -0.3316 |
| NDUFA7 | 0.04031457 | -0.3793683 |
| GZF1 | 0.04091086 | -0.2745701 |
| PDCL | 0.04245787 | -0.2803202 |
| CCDC51 | 0.04318786 | -0.2952535 |
| MT-ND4 | 0.04695626 | -0.6759886 |
| DEDD | 0.04699614 | -0.2218534 |
| DSTN | 0.04760566 | -0.238492 |
| DCTN2 | 0.04838837 | -0.182193 |
| TRAPPC2L | 0.04892909 | -0.4731658 |
| EDARADD | 0.04985603 | -1.4593028 |
